# Evaluation of Marine Agarose Biomaterials for Tissue Engineering Applications

**DOI:** 10.3390/ijms22041923

**Published:** 2021-02-15

**Authors:** Ainhoa Irastorza-Lorenzo, David Sánchez-Porras, Olimpia Ortiz-Arrabal, María José de Frutos, Emilio Esteban, Javier Fernández, Agustín Janer, Antonio Campos, Fernando Campos, Miguel Alaminos

**Affiliations:** 1 Tissue Engineering Group, Department of Histology, University of Granada and Instituto de Investigación Biosanitaria ibs.GRANADA, E18016 Granada, Spain; ainhoairas@gmail.com (A.I.-L.); david.s.p.94@gmail.com (D.S.-P.); olimpiaortiz@correo.ugr.es (O.O.-A.); acampos@ugr.es (A.C.); 2 Hispanagar, SA, E09001 Burgos, Spain; mjfrutos@hispanagar.es (M.J.d.F.); esteban@hispanagar.es (E.E.); javierfdez@hispanagar.es (J.F.); hispanagar@hispanagar.es (A.J.)

**Keywords:** agarose, tissue engineering, biomaterials, biocompatibility, biomechanical properties

## Abstract

Five agarose types (D1LE, D2LE, LM, MS8 and D5) were evaluated in tissue engineering and compared for the first time using an array of analysis methods. Acellular and cellular constructs were generated from 0.3–3%, and their biomechanical properties, in vivo biocompatibility (as determined by LIVE/DEAD, WST-1 and DNA release, with *n* = 6 per sample) and in vivo biocompatibility (by hematological and biochemical analyses and histology, with *n* = 4 animals per agarose type) were analyzed. Results revealed that the biomechanical properties of each hydrogel were related to the agarose concentration (*p* < 0.001). Regarding the agarose type, the highest (*p* < 0.001) Young modulus, stress at fracture and break load were D1LE, D2LE and D5, whereas the strain at fracture was higher in D5 and MS8 at 3% (*p* < 0.05). All agaroses showed high biocompatibility on human skin cells, especially in indirect contact, with a correlation with agarose concentration (*p* = 0.0074 for LIVE/DEAD and *p* = 0.0014 for WST-1) and type, although cell function tended to decrease in direct contact with highly concentrated agaroses. All agaroses were safe in vivo, with no systemic effects as determined by hematological and biochemical analysis and histology of major organs. Locally, implants were partially encapsulated and a pro-regenerative response with abundant M2-type macrophages was found. In summary, we may state that all these agarose types can be safely used in tissue engineering and that the biomechanical properties and biocompatibility were strongly associated to the agarose concentration in the hydrogel and partially associated to the agarose type. These results open the door to the generation of specific agarose-based hydrogels for definite clinical applications such as the human skin, cornea or oral mucosa.

## 1. Introduction

Tissue engineering (TE) is a multidisciplinary field of science whose goal is to generate bioartificial human tissues and organs using cells, scaffolds, bioactive factors and biofabrication technologies [1,2]. Scaffolds play a crucial role in maintaining and controlling cell functions and are the main responsible for the biomechanical properties of the bioartificial tissues [3]. Therefore, biomaterials used as scaffolds in tissue engineering should display adequate biomechanical properties, along with adequate biocompatibility and other characteristics such as bioresorption or biodegradation, adequate internal morphology and cell-friendly fabrication [4,5,6,7].

Naturally-derived polymer biomaterials such as agarose, collagen, chitosan, hyaluronic acid, pectin and fibrin can be directly extracted from plants, animals or human tissues [8,9], and numerous types of biomaterials have been used in different regenerative procedures [10,11,12]. As a number of their components have similar macromolecular structures of native extracellular matrix (ECM), these biomaterials have great potential for use in TE and should trigger a limited inflammatory response [13]. Among other natural polymers, agarose is a promising natural polysaccaryde showing interesting properties for use in TE [14]. Agarose polymers consist of a long chain of agarobiose molecules (1,4-linked, 3-6 anhydro-α-galactose and 1,3-linked-β-D-galactose) that can be extracted from natural marine sources and purified in laboratory. Once purified, agarose has very low content of non-useful oligomers and electronegative groups, is free from toxic and hemolytic substances and shows low degradability by enzymatic hydrolysis. Among its main properties, agarose hydrogels show good transparency, consistent gel strength and thermal hysteresis. Agarose is soluble in water at high temperatures and it jellifies at low temperatures, being a thermal-reversible gel. This phenomenon depends on the molecular weight and concentration of agarose, and the number of side groups [14].

In TE, agarose hydrogels were combined with cells and applied for the regeneration of several hard structures such as the bone [15], cartilage [16] and the inner region of the meniscus [17]. In addition, agarose has been combined with other biomaterials, allowing the generation of biological scaffolds with high versatility towards specific soft tissue types. Among others, agarose biomaterials have been combined with fibrin to generate bioartificial human skin, cornea and oral mucosa [18,19,20], with platelet-rich plasma in cartilage tissue engineering [21], with collagen in the design of vascular endothelial networks [22], and with other biomaterials such as chitosan, graphene oxide and hydroxyapatite for the development of hard tissues [23,24]. Despite the positive results obtained with the use of agarose scaffolds in TE, the biological behavior of these hydrogels still needs further clarification. In addition, different types of agaroses have been obtained during the last years by selecting specific algae clones and extraction methods. As these types of agaroses significantly differ on their gel stiffness as well as hysteresis range [25], and probably also in biocompatibility, their putative usefulness in tissue engineering remains to be elucidated.

The aim of this work was to evaluate the biological and physical properties of five agarose types (D1LE, D2LE, LM, MS8 and D5) as potential biomaterials in TE, by determining their biomechanical properties and biocompatibility, with the ultimate goal of identifying a highly adequate biomaterial for use in the generation of bioartificial tissue-like models by tissue engineering. In this regard, our hypothesis is that some specific types of agarose used at definite concentrations could display more favorable properties than others for use in tissue engineering applications, with the null hypothesis being that no differences exist among these agarose types and concentrations.

## 2. Results

### 2.1. Biomechanical Properties of Agarose Biomaterials

All agarose types and concentrations were able to form a solid hydrogel, except for LM agarose at the lowest concentration of 0.3%, which was not able to form a solid gel supporting the analysis and could therefore not be measured. In general, our analysis of the biomechanical properties of the different acellular agarose hydrogels (AAH) generated in this work revealed that these properties were strongly associated to the agarose concentration in the hydrogel and partially associated to the agarose type.

**Figure 1:**
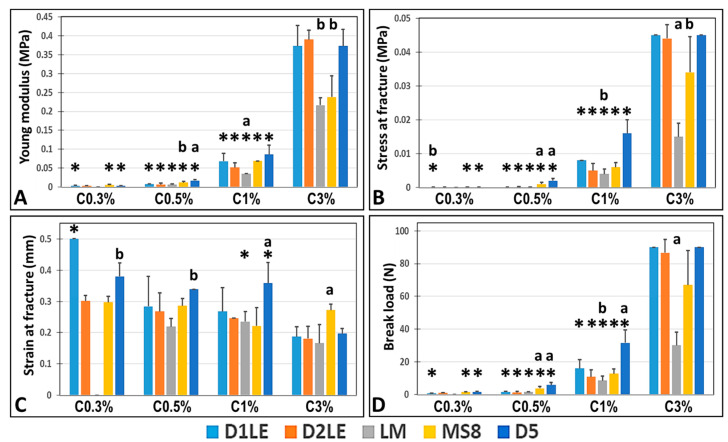
Biomechanical characterization of agarose hydrogels generated with different types of agaroses at increasing concentrations (0.3% to 3%). For each condition, the average values obtained for the Young modulus (panel **A**), stress at fracture (panel **B**), strain at fracture (panel **C**) and break load (panel **D**) are shown. Error bars correspond to standard deviations, since all these measurements were carried out in six-fold (*n* = 6). Statistically significant differences between each specific concentration of each agarose and the next increasing concentration of the same agarose (for instance, D1LE 0.3% vs. D1LE 0.5%) are labeled with asterisks (*). For the comparison among agarose types, specific agarose concentrations showing statistically significant differences with all the other agarose types at the same concentration (for instance, D1LE 0.3% vs. D2LE 0.3%, LM 0.3%, MS8 0.3% and D5 0.3%) are labeled with (^a^), whereas specific concentrations showing statistically significant differences with three of the other agarose types at the same concentration (for instance, D1LE 0.3% vs. D2LE 0.3%, LM 0.3% and MS8 0.3%) are labeled with (^b^).

On the one hand, we found that the concentration of agarose correlated positively with the Young modulus of the AAH (r = 0.5387; *p* < 0.0001), stress at fracture (r = 0.5215, *p* < 0.0001) and break load (r = 0.5149, *p* < 0.0001), and negatively with the strain at fracture (r = −0.5422, *p* < 0.0001). As shown in Figure 1, the highest values of the Young modulus, stress at fracture and break load corresponded to agarose concentrations of 3%, with the highest strain at fracture found for 0.3% agarose (Supplementary Table S1). Statistical global differences among all agarose concentrations (Supplementary Table S2) were found for the Young modulus, stress at fracture, strain at fracture and break load (*p* < 0.0001 for the Kruskal-Wallis test). When agarose concentrations were compared globally within each agarose type, significant differences were found for all parameters for D1LE, D2LE, MS8 and D5, and for the Young modulus, stress at fracture and break load for LM. In addition, global comparison between two specific agarose concentrations showed significant differences for all parameters and all comparisons, except for 0.5% vs. 1% agarose for the strain at fracture (Supplementary Table S2). Then, post-hoc Mann-Whitney comparisons between specific agarose concentrations within each agarose type (Supplementary Table S3) showed statistical differences for the Young modulus, stress at fracture and break load, for all comparisons except for 0.3% vs. 0.5% of each type of agarose. However, comparisons performed for the strain at fracture showed some significant differences among concentrations for all agarose types (Supplementary Table S3).

On the other hand, our analysis of the agarose type showed that this parameter was also related with the biomechanical properties of the AAH, although correlation between agarose type and the different biomechanical parameters was non-significant (*p* > 0.05). In general, the influence of the agarose type was less important than the agarose concentration (Figure 1), and global comparisons among all agarose types using Kruskal-Wallis tests (Supplementary Table S2) only found statistical differences for the strain at fracture. When specific types of agaroses were globally compared regardless the concentration, we found that D5 agarose showed significantly higher strain at fracture than D2LE, LM and MS8, whereas D1LE was higher than LM, and MS8 was higher than LM. In addition, we found statistical differences among all agarose types within each specific concentration of agarose for the stress at fracture, strain at fracture and break load, and for 0.5%, 1% and 3% agarose for the Young modulus (Supplementary Table S2). Post-hoc analyses between specific groups of samples allowed us to detect statistically significant differences that are shown in Supplementary Table S3. In general, the highest differences were found for the 3% concentrations of agarose. For this concentration, D1LE, D2LE and D5 agaroses were significantly higher than LM for the Young modulus, stress at fracture and break load, whereas MS8 was significantly higher than the other agarose types for the strain at fracture. Differences for other agarose concentrations are summarized in Supplementary Table S3.

### 2.2. Indirect Effect on Cell Viability and Function

**Figure 2:**
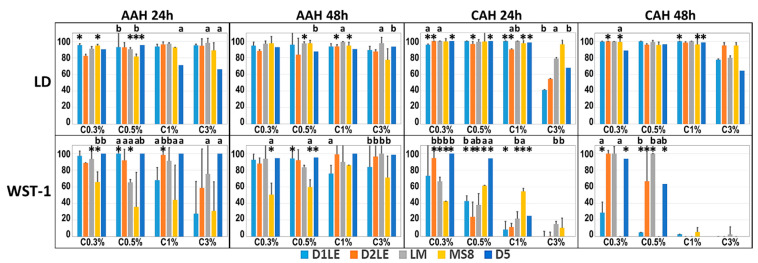
Analysis of cell viability as determined by LIVE/DEAD (LD) and WST-1 in human cells cultured in indirect contact with agarose hydrogels (AAH) and cells dispersed in bioartificial agarose tissue substitutes (cellular agarose hydrogels or CAH) for 24 and 48 h. Results are shown as percentages of LD and WST-1 activity normalized vs. positive controls (cells cultured with FC culture medium without agarose) considered as 100% and negative controls (cells treated with triton X-100) considered as 0%. Error bars correspond to standard deviations, since all these measurements were carried out in six-fold (*n* = 6) with three technical replicates per analysis. Statistically significant differences between each specific concentration of each agarose and the next increasing concentration of the same agarose (for instance, D1LE 0.3% vs. D1LE 0.5%) are labeled with asterisks (*). For the comparison among agarose types, specific agarose concentrations showing statistically significant differences with all the other agarose types at the same concentration (for instance, D1LE 0.3% vs. D2LE 0.3%, LM 0.3%, MS8 0.3% and D5 0.3%) are labeled with (^a^), whereas specific concentrations showing statistically significant differences with three of the other agarose types at the same concentration (for instance, D1LE 0.3% vs. D2LE 0.3%, LM 0.3% and MS8 0.3%) are labeled with (^b^).

In general, we found that most agarose types and concentrations were associated with high levels of cell viability and functionality when human cells were cultured in indirect contact with AAH for 24 h and 48 h. First, we analyzed the percentage of dead and live cells using the LD assay. As shown in Supplementary Table S1 and Figure 2, most cell cultures were viable, with a percentage of live cells above 70% for all agarose types and concentrations except 3% D5 agarose. We found a significant correlation between the percentage of live cells and the agarose concentration (r = −0.0973, *p* = 0.0295), and with the agarose type (r = 0.1174, *p* = 0.0074). Global comparisons among all agarose concentrations were non-significant at 24 h, and only showed significant differences between 0.3% vs. 3% agarose and 1% vs. 3% agarose at 48 h. However, analysis of the agarose type showed significant differences at 24 and 48 h, with the highest cell viability found for LM, followed by D1LE. Differences among agaroses were significant for 0.5%, 1% and 3% agarose at both study times and 0.3% at 48 h, and pair-wise comparisons between agarose types regardless the concentration was significant at 24 or 48 h for most comparisons except for D1LE vs. D2LE. Statistical differences between specific groups of samples are shown in Supplementary Table S3.

For the metabolic assay WST-1, we found that the physiological activity of the human cells cultured in indirect contact with AAH was high in most groups (Supplementary Table S1 and Figure 2), with a significant correlation with the agarose concentration (r = −0.0983, *p* = 0.0014) and type (*p* < 0.0001, r = 0.1246). All global groups of samples showed at least 70% of functional activity, except for MS8, for the global groups of agarose types, and 3% agarose at 24 h for the concentration global groups. Statistical differences among agarose types were found at both study times, and most differences among concentrations were found at 24 h (Supplementary Table S2). For the specific groups, we found that the lowest metabolic activity levels mostly corresponded to the highest agarose concentrations at 24 h of culture, with less differences found after 48 h of culture (Supplementary Table S3).

Finally, we quantified the cell DNA released from cells cultured in indirect contact with AAH. Results showed that all study groups were associated to very low amounts of DNA (nearly 0 in all cases) (Supplementary Table S1), with no correlation with the agarose concentration or type (*p* > 0.05). No differences were found among agarose concentrations or types (Supplementary Tables S2 and S3).

### 2.3. Direct Effect on Cell Viability and Function

Analysis of the viability of cells cultured in direct contact with agarose biomaterials using LD assays demonstrated that most conditions were biocompatible (Figure 2). A significant correlation was found between cell viability and the concentration of agarose used in each cellular agarose hydrogel (CAH) (r = −0.4200, *p* < 0.001), but not with the type of agarose (*p* > 0.05). Statistical comparisons among all agarose concentrations within each agarose type were statistically significant at both study times (Supplementary Table S2). Particularly, we found that 3% agarose showed significantly lower viability than the other concentrations (Supplementary Table S3). Regarding the agarose type, global comparisons were significant at 48 h, and differences among agarose types were found within each concentration (Supplementary Tables S1 and S2). At 24 h, D2LE showed lower viability than LM, whereas MS8 showed some global differences with D1LE, D2LE and D5 at 48 h. For the highest concentration of agarose, the highest viability corresponded to MS8 agarose at both study times, with the lowest values found for D1LE and D2LE agarose at 24 h. Statistical differences were found for some specific comparisons (Supplementary Table S3)

Regarding the functional WST-1 assay, our results showed a significant correlation between cell function and the agarose concentration (r = −0.5750, *p* < 0.001), but non-significant (*p* > 0.05) regarding the agarose type. In general, the highest agarose concentrations were associated to lower values of WST-1 activity at both study times (Supplementary Table S1 and Figure 2), with a significant difference among all agarose concentrations for the global comparisons (Supplementary Table S2). Indeed, comparisons of agarose concentrations regardless the agarose type were statistically significant for all comparisons except for 0.3% vs. 0.5% and 1% vs. 3% agarose at 48 h, and significant differences among concentrations were found within each type of agarose (Supplementary Table S2). Supplementary Table S3 shows statistical differences for some specific comparisons, with 1% and 3% agarose showing the lowest WST-1 values for all agarose types. In general, the highest WST-1 values corresponded to the lowest agarose concentrations, especially for agarose D5 at 24 h and D2LE, LM and D5 at 48 h. Global comparisons among agarose types were significant at both study times, and differences among agarose types were found for each agarose concentration at 24 h, and for 0.3% and 0.5% agarose at 48 h. Differences among agarose types are summarized in Supplementary Tables S2 and S3.

As in the case of cells cultured in indirect contact with agarose, cells immersed within the CAH showed very little DNA released as a consequence of cell death, with values below 4% mortality for all conditions (Supplementary Table S1). Despite these very low values, correlation with the agarose concentration was significant (r = −0.2607, *p* < 0.001), and some statistical differences among agarose types and concentrations were found (Supplementary Tables S2 and S3).

### 2.4. In Vivo Biocompatibility

**Figure 3:**
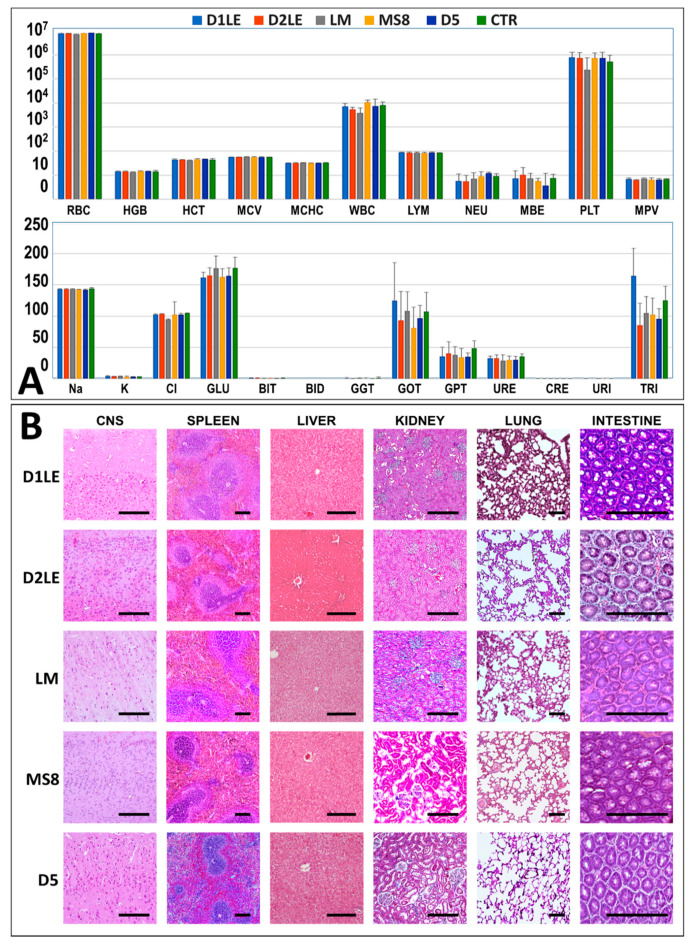
In vivo biocompatibility analysis of the different agarose types (D1LE, D2LE, LM, MS8 or D5) grafted in laboratory rats and controls. (**A**): Average values of the main hematological and biochemical parameters analyzed in blood. Hematological parameters analyzed here included red blood cells (RBC), hemoglobin (HGB), hematocrit (HCT), mean cell volume (MCV), mean red blood cell hemoglobin content (MCHC), white blood cells (WBC), percentage of lymphocytes (LYM), percentage of neutrophils (NEU), percentage of monocytes–basophils–eosinophils (MBE), percent-age of platelets (PLT) and mean platelet volume (MPV). Biochemical parameters included: sodium (Na), potassium (K), chlorine (Cl), glucose (GLU), total bilirubin (BIT), direct bilirubin (BID), gamma-glutamyl transpeptidase (GGT), aspartate aminotransferase (GOT), alanine aminotransferase (GPT), urea (URE), creatinine (CRE), uric acid (URI) and triglycerides (TRI). CTR: Control animals devoid of grafted biomaterials. Error bars correspond to standard deviations, since all these measurements were carried out in four-fold (*n* = 4). (**B**): Histological analysis of major organs of each animal: central nervous system (CNS), spleen, liver, kidney, lung and intestine. Scale bars: 200 µm.

To determine the biocompatibility of the different types of agarose, AAH were grafted in laboratory animals for 3 months. Analysis of the main hematological and biochemical parameters in blood (Figure 3A) showed no significant modifications of any of the analyzed parameters, with all parameters being similar in all study animals and controls. In addition, the histological analysis of major distal organs of animals grafted with the different agarose types (Figure 3B) revealed that the structure of the central nervous system, spleen, liver, kidney, lung and intestine was normal and devoid of any histological alteration in all study animals. No signs of necrosis, infection, rejection or other histological alterations were detected, and no differences were found among animals grafted with the different agarose types.

**Figure 4:**
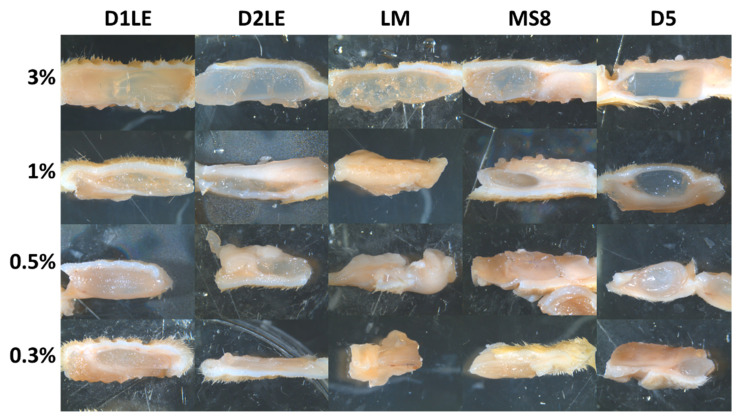
Macroscopical analysis of the biomaterials subcutaneously grafted for 3 months in each animal (*n* = 4 per group). For each agarose type and concentration, the gross aspect of the implant site is shown.

To determine the local effects of the different agaroses grafted subcutaneously, we first analyzed the grafting area at the macroscopical level (Figure 4). Results showed no clinical signs of rejection, infection, hemorrhage or other pathological signs. All agarose types at the concentration of 3% were very well preserved after 3 months of follow-up, with some signs of fragmentation only for LM and MS8 agaroses. For the rest of concentrations, the biomaterial was detectable in most cases, except for the 0.3% concentration of D2LE, LM and MS8 agaroses.

**Figure 5:**
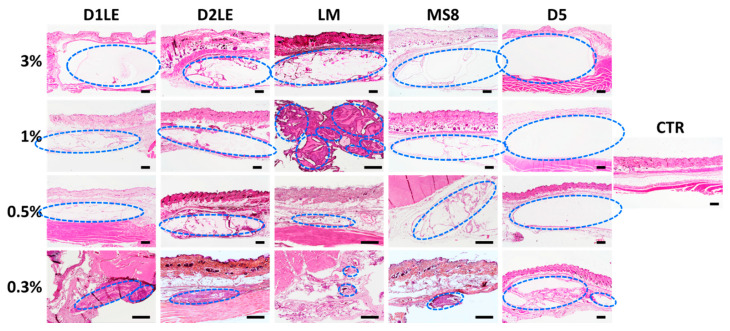
Histological analysis of the grafting site of each animal after 3 months of follow-up using hematoxylin-eosin (HE) staining. Each animal was grafted with different concentrations (3%, 1%, 0.5% and 0.3%) of each agarose type (D1LE, D2LE, LM, MS8 or D5). CTR: control non-grafted animals. Areas surrounded by blue dotted circles correspond to agarose biomaterials. Four animals were included in each group (five agarose types and controls). Scale bars: 500 µm.

**Figure 6:**
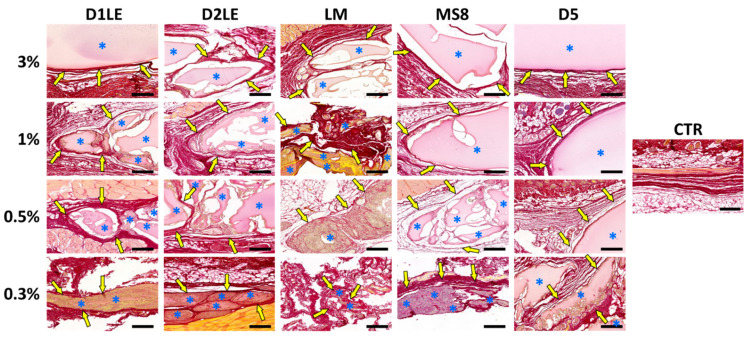
Histochemical analysis of the grafting site of each animal after 3 months of follow-up using picrosirius red (for collagen fibers detection). Each animal was grafted with different concentrations (3%, 1%, 0.5% and 0.3%) of each agarose type (D1LE, D2LE, LM, MS8 or D5). CTR: control non-grafted animals. Areas corresponding to agarose biomaterials are labeled with asterisks (*) and the host connective tissue forming a thin layer surrounding and encapsulating the biomaterial are highlighted with arrows. Four animals were included in each group (five agarose types and controls). Scale bars: 500 µm.

Then, we analyzed the microscopical structure of the grafting area to determine the effects of each biomaterial at the structural level. As shown in Figure 5, all agarose types demonstrated to be highly biocompatible, since none of the samples showed any microscopical signs of rejection, infection, tumorigenesis or necrosis. Analysis of samples stained with hematoxylin and eosin confirmed the presence of each biomaterial at the grafting site, especially at the highest concentrations of agarose. Interestingly, we found that the host connective tissue formed a thin layer surrounding and encapsulating each biomaterial, and this tissue emitted some connective tissue prolongations able to penetrate into the biomaterial, especially for the lowest agarose concentrations. These connective extensions were especially abundant in agarose LM and, partially, in D2LE and MS8, whereas D5 was mostly devoid of them, except for the lowest concentrations. In order to determine if the connective tissue surrounding the biomaterial was fibrotic, we stained each sample with picrosirius red (Figure 6). Results showed that the intensity of collagen fibers at the graft site was comparable to control tissues, and none of the samples showed any signs of pathological fibrosis.

**Figure 7:**
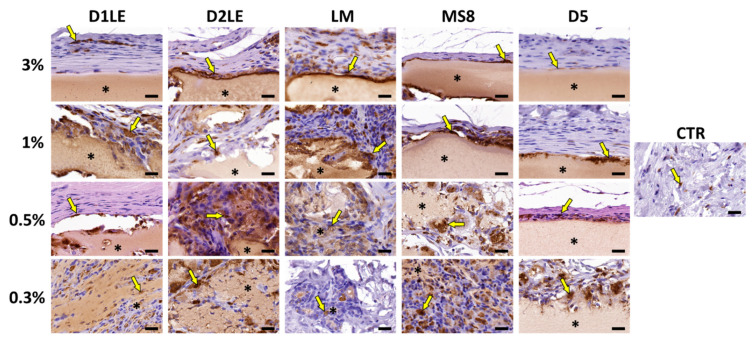
Immunohistochemical analysis of macrophage cells at the grafting site of each animal after 3 months of follow-up using anti-CD68 primary antibodies. Each animal was grafted with different concentrations (3%, 1%, 0.5% and 0.3%) of each agarose type (D1LE, D2LE, LM, MS8 or D5). CTR: control non-grafted animals. Illustrative areas corresponding to agarose biomaterials are labeled with asterisks (*) and some CD68-positive cells (brown cells) are highlighted with arrows. four animals were included in each group (five agarose types and controls). Scale bars: 20 µm.

**Figure 8:**
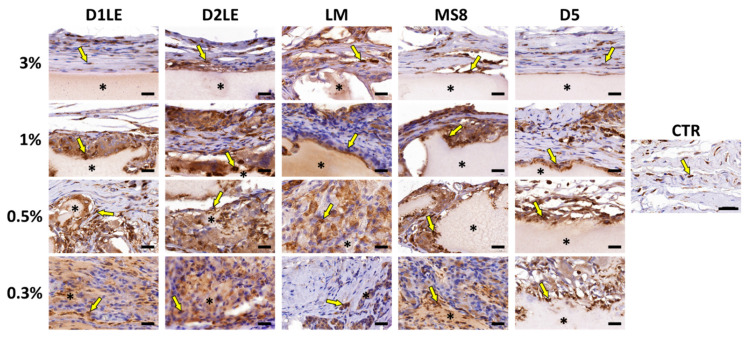
Immunohistochemical analysis of pro-regenerative M2 macrophage cells at the grafting site of each animal after 3 months of follow-up using anti-CD206 primary antibodies. Each animal was grafted with different concentrations (3%, 1%, 0.5% and 0.3%) of each agarose type (D1LE, D2LE, LM, MS8 or D5). CTR: control non-grafted animals. Illustrative areas corresponding to agarose biomaterials are labeled with asterisks (*) and some CD206-positive cells (brown cells) are highlighted with arrows. 4 animals were included in each group (five agarose types and controls). Scale bars: 20 µm.

**Figure 9:**
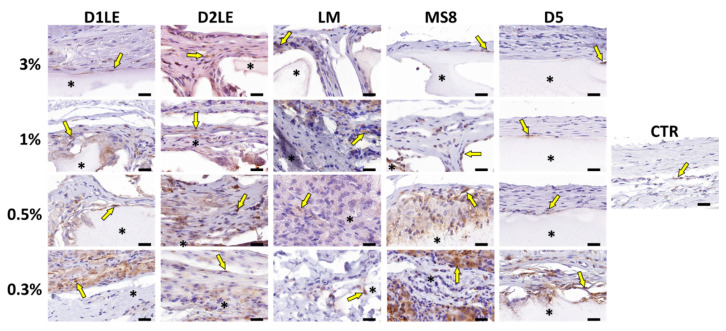
Immunohistochemical analysis of inflammatory CD4-positive cells (mostly, T helper lymphocytes) at the grafting site of each animal after 3 months of follow-up. Each animal was grafted with different concentrations (3%, 1%, 0.5% and 0.3%) of each agarose type (D1LE, D2LE, LM, MS8 or D5). CTR: control non-grafted animals. Illustrative areas corresponding to agarose biomaterials are labeled with asterisks (*) and some CD4-positive cells (brown cells) are highlighted with arrows. 4 animals were included in each group (5 agarose types and controls). Scale bars: 20 µm.

**Figure 10:**
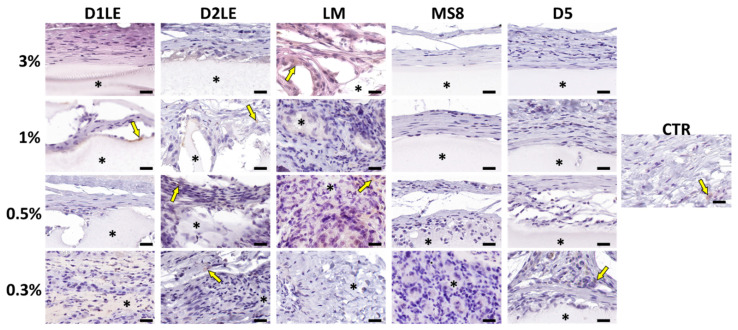
Immunohistochemical analysis of inflammatory CD8-positive cells (mostly, T cytotoxic lymphocytes) at the grafting site of each animal after 3 months of follow-up. Each animal was grafted with different concentrations (3%, 1%, 0.5% and 0.3%) of each agarose type (D1LE, D2LE, LM, MS8 or D5). CTR: control non-grafted animals. Illustrative areas corresponding to agarose biomaterials are labeled with asterisks (*) and some CD4-positive cells (brown cells) are highlighted with arrows. Four animals were included in each group (five agarose types and controls). Scale bars: 20 µm.

To determine if the different agarose types are able to trigger an inflammatory reaction at the graft site, specific immune system cells were identified using specific antibodies. As shown in Figure 7, Figure 8, Figure 9 and Figure 10, we found that most inflammatory cells recruited at the grafting site were macrophages (CD68-positive cells, Figure 7), and that most of these macrophages were CD206-positive pro-regenerative M2 macrophages (Figure 8). In contrast, the number of cells detected by CD4 (Figure 9) and CD8 (Figure 10) immunohistochemistry -mostly, T helper and cytotoxic T cells, respectively- was very low, especially, in the case of CD8.

## 3. Discussion

Agarose hydrogels have been previously used in TE due to their capability to reproduce the macromolecular structure of native ECM and their high capacity to absorb water, nutrients and oxygen, allowing cell adhesion, proliferation and differentiation [14]. In general, agarose biomaterials display temperature-dependent gelling capability and tunable biomechanical properties [26] that strongly depend on the concentration of agarose and water in the biomaterial [14,26,27]. The fact that these factors are controllable during the biofabrication process, makes possible the generation of different types of scaffolds with definite biomechanical properties for use in TE [27]. Recent advances in the field allowed the generation of different agarose types differing in their molecular structure, gel strength and hysteresis range. However, most of the recently synthetized agaroses types have not been characterized to determine their potential usefulness in TE. For the first time, five different agaroses were evaluated and compared as putative biomaterials for the construction of bioartificial human tissues by TE. In this regard, we analyzed five different agarose types that were not previously tested and compared in tissue engineering at the ex vivo and in vivo levels by using an array of methods and techniques to determine their biomechanical properties and biocompatibility.

First, we characterized each agarose hydrogel to determine the influence of the concentration and type of agarose on the biomechanical properties of these biomaterials. As expected [27,28], we found that the concentration of agarose was the main factor affecting the biomechanical properties of the hydrogels, and highly concentrated hydrogels showed the highest values of Young modulus. In general, agarose biomaterials were more elastic than viscous, and the Young modulus of 3% agarose hydrogels was more than 50-fold higher than 0.3% biomaterials. As the biomechanical properties of hydrogels cannot be fully described by a single parameter, characterization was achieved using other types of measurements [3]. In this regard, we also analyzed the stress at fracture, break load and strain at fracture, and we found a correlation with the Young modulus. In consequence, these results confirm that the biomechanical properties of the agarose hydrogels can be adjusted within a broad range by modifying the concentration of agarose to adjust these properties to specific native tissues [27,28]. 

Although at lower levels, we found that the agarose type also influenced the biomechanical properties of the hydrogels evaluated in this work. In general, the agarose type was directly associated to the strain at fracture. This parameter is one of the major determinants of the elastic behavior of the biomaterial, as it represents the deformation percentage of the hydrogel at the moment of fracture [29]. Therefore, our results imply that the use of specific agarose types is associated to hydrogels with different elastic behavior and suggest that different types of agarose could be used to obtain biomaterials with definite biomimetic elastic behavior. Although further research is in need, these results suggest that the agaroses showing highly elastic properties, especially D5 at most concentrations and MS8 at the highest concentration, should be preferentially used for the generation of human tissues and organs displaying an elastic behavior in vivo, such as the human skin [30] and rectal tissue [31]. On the other hand, we found that the type of agarose significantly influenced the stiffness of the agarose hydrogels at certain concentrations. Specifically, we found that agaroses D1LE, D2LE and D5 showed higher stiffness than LM and MS8 at the highest concentration of agarose, suggesting again that the biomechanical properties of the agarose hydrogels can be controlled during the biofabrication process. In consequence, we would recommend the use of the stiffer agaroses D1LE, D2LE and D5 for the generation of human organs requiring higher rigidity such as the human vagina [31] or the articular cartilage [32].

Along with the biomechanical behavior, biocompatibility is a requirement of biomaterials used in TE. In this regard, we analyzed the effect of each agarose type and concentration on human cells using different methods and techniques. This comprehensive ex vivo approach allowed us to determine cell viability and function with high accuracy, as previously demonstrated [33]. In general, our results demonstrated that the different agarose types were able to support cell physiology with no relevant side effects on cells cultured in the presence of agarose. First, the DNA quantification analysis allowed us to demonstrate that agarose -in direct or indirect contact with cells- did not induce an intense process of cell death with membrane disruption. Then, we used LD method to identify cell membrane damage and cytoplasmic enzymes function. Results obtained with this highly sensitive combined method showed an average cell viability above 90% at 24 and 48 h for the direct and indirect contact study. However, we found that the highest concentrations of agarose were associated to a reduction in cell activity, especially for cells cultured within the biomaterial of the direct effect study, with very few differences among agarose types. These results are in agreement with previous works demonstrating that biomaterial stiffness plays a crucial role in regulating cell fate, adhesion, migration and differentiation [34,35]. Finally, ex vivo biocompatibility was determined by analyzing the effects of each agarose type and concentration on mitochondrial cell metabolism through WST-1 assays. Results showed that the indirect contact with cells was not associated to a severe impairment of these cell functions in most of the cases. However, when cells were cultured within the biomaterial, we found a clear decrease of mitochondrial cell metabolism, especially for the highest concentrations of agarose. An interesting question is whether or not the cells can actively proliferate once embedded in agarose biomaterials. In this regard, our results demonstrated a significant decrease of WST-1 activity at the highest agarose concentrations, especially after 48 h of follow-up. Although proliferation was not directly assessed, it is likely that cells cannot proliferate within very dense biomaterials. Again, the essential role of the biomechanical properties of the scaffold on key cell functions could explain these results [34,35], and suggest that agarose biomaterials should be used at the lowest possible concentrations. In fact, the clear reduction in metabolic activity found at the highest agarose concentrations and follow-up times may imply that highly concentrated agaroses should preferably be used as acellular scaffolds, as suggested for the human cartilage [36]. However, bioengineered tissues requiring an abundant cell population such as the human skin and cornea [3,19,37], should be used at the agarose concentrations showing the highest metabolic activity such as D2LE, LM and D5 agaroses at the concentration of 0.5%. Future experiments should determine the most proper conditions for the generation of cellular agarose-based biomaterials by tissue engineering.

Once biocompatibility was assessed ex vivo, we analyzed the in vivo effects of each agarose type as a critical phase of the preclinical evaluation of bioengineered tissues for future clinical use [18]. Our results first showed that animals grafted with the different agarose biomaterials were devoid of any detectable systemic alteration as determined by a wide range of biochemical markers analyzed in blood, including some specific parameters of hepatic, renal and metabolic functions. These results are in agreement with our histological analysis of six major organs, which demonstrated that the structural pattern of the central nervous system, spleen, liver, kidney, lung and intestine was normal in all animals grafted with the different biomaterials. It is well-known that all these parameters are often altered in animals affected by severe diseases, such as traumatic injuries, cancer or immune rejection [38,39]. In general, all these results suggest that implantation of the different agarose hydrogels was safe for the animals and was not associated to rejection, infection, tumorigenesis or organ necrosis at the systemic level. In the second place, we analyzed the grafting site to determine the local effects of each grafted biomaterial. In agreement with the systemic analysis, we found that the grafting site was devoid from any detectable alteration, and most biomaterials appeared to remain unaltered at the local area after 3 months of follow-up, especially for the highly concentrated hydrogels. In general, we found that the most concentrated agaroses were efficiently encapsulated by a non-fibrotic connective tissue surrounding and enveloping the biomaterial, and most host immune cells were not able to penetrate into the biomaterial. These results are in agreement with previous works suggesting that agarose biomaterials are difficult to degrade and remain at the graft site [14,40]. However, the lowest concentrations of agarose resulted in the host tissue penetrating into the hydrogels, with abundant immune system cells trying to engulf and remodel the grafted biomaterial. Interestingly, differences were found among the five agarose types, and the agaroses showing the lowest stiffness values -LM and MS8- showed a faster remodeling rate. In consequence, we may state that the low degradability of agarose biomaterials is only applicable to highly concentrated hydrogels, which probably show low porosity, whereas low concentrations of each agarose and agaroses with the lowest stiffness, would behave as degradable biomaterials, at least at the follow-up times analyzed here. These in vivo results are in agreement with our previous ex vivo findings suggesting that cells in direct contact with highly concentrated agaroses may not be fully functional. Although a detailed analysis of the molecular structure and pore size of each agarose type and concentration is in need, we could hypothesize that highly dense agaroses could not allow cells to easily migrate and spread within the biomaterial, thus preventing biodegradation and remodeling of this type of biomaterials in a short period of time. Again, the in vivo results support our hypothesis that the use of highly concentrated agaroses could be indicated in cases requiring long-term stability and low cellularity, such as the human intervertebral disk [41]. However, other tissues and organs with an abundant cell population requiring a progressive remodeling and biodegradation, such as the human skin and cornea [3,19,37], should use low concentrations of agarose. Although further studies are in need in this regard, this would be applicable not only to orthotypical cells obtained from the tissue to be reproduced in laboratory, but also to alternative cell sources like human mesenchymal stem cells (MSC) and other cell types, as previously suggested [42,43]. 

Several previous reports demonstrated that in vivo grafted biomaterials can trigger either a pro-inflammatory response with abundant fibrosis and high expression of pro-inflammatory cytokines, activated CD4-positive T helper cells and M1 macrophages, or a pro-regenerative response characterized by abundant M2 macrophages and scarce lymphocytes [44,45,46]. In our case, the five types of agaroses demonstrated to be able to induce a pro-regenerative reaction in all agarose types and concentrations, suggesting that these biomaterials were highly biocompatible [47]. 

In conclusion, in the present work, we evaluated and characterized five types of agaroses for use in TE. Although all agarose types were highly biocompatible, we found that each agarose showed specific properties for use in TE. In general, we propose that tissues requiring highly stiff biomaterials that should remain at the grafting site for a long time, such as the human bone or cartilage, should use the highest concentrations of agaroses displaying the highest stiffness such as D1LE, D2LE or D5. Due to the difficultness of cells to exert their functions immersed within dense biomaterials, highly concentrated agarose hydrogels should tend to keep the cell population seeded inside as low as possible. However, other tissues requiring highly elastic biomaterials and a more rapid biodegradation such as the human skin, cornea or rectal tissue, should use low concentrations of the most elastic agaroses such as D5 or MS8. In these cases, agarose hydrogels could be populated with cultured human cells without significantly altering cell function and metabolism, which is a requirement for this type of cell-rich tissues and organs. Although future studies should determine the exact usefulness of each biomaterial for specific TE applications, the novelty of the study is that our results allowed us to identify particular agarose types and concentrations for definite uses in TE using an array of ex vivo and in vivo techniques and methods.

The present study has several limitations that should be addressed in future works. First, a complete structural and molecular characterization of the different agarose types and concentrations is still in need, including a detailed analysis of pore size, water retention, surface rugosity and other physical and chemical properties. Then, a further study should be carried out using a larger cohort of samples and animals. Once this exploratory study was performed, future studies should be performed after determining the sample size, including a power analysis to evaluate the differences among each agarose type and concentration with higher statistical power. Finally, future in vivo studies should be carried out using polyethylene tubes or other systems allowing an efficient and exact control of the amount and shape of the biomaterials grafted in each animal, as previously suggested [48]. Future perspectives also include the need of characterize the different agarose types according to the guidelines of the European Medicines Agency for advanced therapies before clinical use.

## 4. Materials and Methods

### 4.1. Materials

Materials and reagents used in this work are summarized in Supplementary Table S4.

### 4.2. Human Cell Cultures

Human skin biopsies were obtained from healthy donors subjected to minor surgery under local anesthesia. Biopsies were washed in phosphate buffered saline (PBS) and enzymatically digested with type-I collagenase as previously described [19]. Isolated skin fibroblasts were cultured and expanded using a culture medium consisting of Dulbecco’s Modified Eagle Medium (DMEM) supplemented with 1% antibiotic cocktail solution and 10% fetal bovine serum (all these materials, from Sigma-Aldrich (St Louis, MO, USA) under standard cell culture conditions. This work was approved by the local ethics research committee (ref. 0022-N-19) and all skin donors provided informed consent for their participation in the study. 

### 4.3. Generation of Agarose Hydrogels

In this study, 5 types of agarose with different chemical and physical properties, were evaluated (D1LE, D2LE, LM, MS8 and D5, Hispanagar, Burgos, Spain). All these five agaroses were obtained from different species of agarophyte seaweeds harvested at different geographical areas, and different processing protocols were applied for the generation of each agarose type. Specifically, D1LE, D2LE and D5 correspond to native agaroses extracted from different kinds of red algae, whereas LM and MS8 are derivatized materials subjected to additional chemical modifications resulting in different physical and chemical properties. Briefly, D1LE can be considered as a routine agarose showing a hysteresis of 36.6–88.3 °C and a gel strength at 1.5% of 2990 g/cm^2^; D2LE tends to jellify faster than the other agarose types, and it has a hysteresis of 40.6–87.8 °C and a gel strength at 1.5% of 2310 g/cm^2^; LM has very low gelling temperature as a result of derivatization, and has a hysteresis of 26.1–64.9 °C and a gel strength at 1% of 1100 g/cm^2^; MS8 was subjected to a derivatization process resulting in smaller pore size. and has a hysteresis of 34–78.5 °C and a gel strength at 1.5% of 3590 g/cm^2^; and D5 is able to generate stiffer hydrogels and has a hysteresis of 36.2–88.1 °C and a gel strength at 1.5% of 4120 g/cm^2^.

Acellular agarose hydrogels (AAH) were prepared at four different concentrations (0.3%, 0.5%, 1% and 3%) in sterile conditions. First, a stock solution was prepared from each agarose type by diluting each agarose in PBS (Sigma-Aldrich) at a final concentration of 4%. Then, this stock solution was melted and diluted in PBS to the desired final concentration of agarose, poured into culture plates (Corning, New York, NY, USA) and allowed to jellify at 37 °C.

In addition, bioartificial agarose tissue substitutes consisting in cellular agarose hydrogels (CAH) were generated by culturing human skin fibroblasts within agarose biomaterials in order to generate cellular constructs in which cells contacted directly with the biomaterial. Briefly, different types of agarose biomaterials were generated as described above, and 2 × 10^4^ cultured human skin fibroblasts were added per mL of hydrogel when the mixture reached 37 °C, just before jellification in culture plates. These tissue substitutes were kept at 37 °C in a cell culture incubator with 5% CO_2_. The culture medium was renewed every three days.

### 4.4. Analysis of Biomechanical Properties

Biomechanical properties of the different samples generated in this work were assessed using an Instron Model 5943 biomechanical analyzer (Norwood, MA, USA) with a BlueHill 3 software by compression testing. 6 cm-diameter and 5 mm-thickness AAH were generated with the different agarose types and concentrations. Briefly, AAH were carefully placed between the two plates, and a progressive compression force was applied by the upper plate using a load cell of 100 N with a ramp displacement of 0.005 mm/s. All samples were measured using the same environmental conditions and all analyses were carried out in six-fold (*n* = 6).

### 4.5. Ex Vivo Analysis of Biocompatibility, Cell Viability and Function

The biological effect of the different types of agaroses on human cells was determined using a direct and an indirect cell-agarose contact analysis based on the recommendations of the ISO 10993-5: 2009. For the indirect contact analysis, 10^4^ cells were seeded on culture wells. Then, 6.5 mm-diameter porous inserts (Corning) filled with 500 µL of AAH were placed on top of each culture well, with the culture medium contacting the agarose and the cells. For the direct contact analyses 2 × 10^4^ cells/mL were embedded in the agarose biomaterial during the preparation of each hydrogel, and samples of 500 µL of volume were placed in culture wells and allowed to jellify at 37 °C with 5% CO_2_ in an incubator. Then, three analysis methods were used to determine cell viability and function after 24 and 48 h as described below.

In order to determine the biological effect of the different agaroses on cell membrane and cytoplasmic enzymes function, LIVE/DEAD (LD) cell viability/cytotoxicity analysis kit (Life Technologies, Carlsbad, CA, USA) was used following the manufacturer’s recommendations [49,50]. To begin, cells were washed with PBS and incubated for 15′ in a working solution consisting in acetomethoxycalcein and ethidium bromide in PBS. Then, the solution was removed, samples were washed with PBS and viable (green fluorescence) and dead (red fluorescence) cells were identified using an Eclipse Ti-U inverse fluorescent microscopy (Nikon, Tokyo, Japan). Cell viability was calculated as the percentage of live cells from the total cell number (live and dead cells).

For the functional analysis of mitochondrial cell metabolism, WST-1 colorimetric assay was used (Cell Proliferation Reagent WST-1, Sigma Aldrich, St. Louis, MO, USA) as previously described [49,50]. Briefly, water-soluble tetrazolium salt was added to the culture medium and after 4 h incubation absorbance was read with a ASYS UVM340 microplate reader (Biochrom, Cambridge, UK).

Finally, the cytotoxicity was determined by quantification of free DNA released by dead cells to the culture medium, we used previously described methods [49,51]. In brief, the culture medium was obtained from wells of each study group and a UV-Vis NanoDrop 2000 equipment (Thermo Fisher Scientific, Waltham, MA, USA) was used to measure total DNA present in the medium. 

For the three analysis methods, positive and negative controls were used at each study time. For WST-1 and DNA, results were normalized based on the results obtained for positive and negative controls and expressed as percentage of activity compared to positive controls of live cells. Positive controls consisted in human cells cultured on cell culture plates with FC culture medium. As negative controls, cultured cells were treated with triton X-100 (Sigma-Aldrich) to induce cell death. All analyses were carried out in six-fold with three technical replicates per analysis.

### 4.6. In Vivo Analysis

In vivo biocompatibility of the different types of agaroses was evaluated by subcutaneous implantation in 12 weeks old Wistar rats following the ISO 10993-6: 2017. AAH were generated in sterility at each concentration (0.3%, 0.5%, 1% and 3%) of each agarose type (D1LE, D2LE, LM, MS8 and D5). In order to ensure that the size and volume of each implant were identical for all animals, AAH were generated in 24-well culture plates (TC-Treated Multiple Well Plates, Corning) with a well area of 1.9 cm^2^ using a final volume of 1 mL of agarose mixture per well. Once jellified, each agarose disk was divided in two identical halves of 0.5 mL of volume that were grafted in each animal.

Then, animals were deeply anesthetized with ketamine and acepromazine (both from Boehringer Ingelheim, Ingelheim am Rhein, Germany) and each rat was subcutaneously grafted with one type of agarose at different concentrations (0.3%, 0.5%, 1% and 3%), with the agarose type being randomly assigned to each animal. Each implant was separated at least 3 cm from the other implants. A total of 24 animals were used in the present study (4 animals grafted with each agarose type and 4 non-grafted control animals). Experiments were performed according to the European Union and Spanish Government guidelines for the ethical care of animals (EU Directive No. 63/2010, RD 53/2013). Animal experimentation and the research work were approved by the local ethical committee (refs. 03-7-15-311 and 08-07-2019-121).

After 3 months of follow-up, animals were euthanatized under general anesthesia. Peripheral blood was collected at the moment of sacrifice and relevant hematological as well as biochemical parameters were determined by using a Sysmex KX-21N automatic analyzer and a clinical chemistry analyzer Cobas c311 (both from Roche, Basel, Switzerland). Hematological parameters analyzed here included red blood cells (RBC), hemoglobin (HGB), hematocrit (HCT), mean cell volume (MCV), mean red blood cell hemoglobin content (MCHC), white blood cells (WBC), percentage of lymphocytes (LYM), percentage of neutrophils (NEU), percentage of monocytes–basophils–eosinophils (MBE), percentage of platelets (PLT) and mean platelet volume (MPV). Biochemical parameters included: sodium (Na), potassium (K), chlorine (Cl), glucose (GLU), total bilirubin (BIT), direct bilirubin (BID), gamma-glutamyl transpeptidase (GGT), aspartate aminotransferase (GOT), alanine aminotransferase (GPT), urea (URE), creatinine (CRE), uric acid (URI) and triglycerides (TRI).

### 4.7. Histological Analysis

At the moment of the euthanasia, tissues corresponding to the local implantation sites and several major organs (central nervous system, spleen, liver, kidney, lung and intestine) were collected and macroscopical images were taken from each implantation site. Tissues were fixed in buffered formalin and embedded in paraffin (both from Panreac Química S.L.U., Barcelona, Spain) following routine histological methods. 4 µm-thick sections were obtained, dewaxed in ethanol series, rehydrated and stained with hematoxylin and eosin (HE) (Panreac Química S.L.U.) using standard protocols. The presence of mature collagen fibers was evaluated by Picrosirius histochemistry using Sirius red F3B (Sigma-Aldrich) for 30 min as previously described [19,52]. Detection of specific immune system cells was performed by immunohistochemistry using specific antibodies and standard protocols. Briefly, tissue sections were dewaxed and rehydrated, and antigen retrieval was performed using pH 8 EDTA (for CD4 and CD8) or pH 6 citrate (for CD68 and CD206) (Sigma-Aldrich). Endogenous peroxidases were quenched with H_2_O_2_ (Panreac Química S.L.U.) and prehybridization was performed with a solution containing casein and horse serum (Vector laboratories, Burlingame, CA, USA). Then, samples were incubated overnight with one of the following primary antibodies (all of them from Abcam, Cambridge, UK): rabbit monoclonal anti-CD4 (ref. ab237722, dilution 1:4000), rabbit monoclonal anti-CD8 (ab237709, dilution 1 µg/mL), mouse monoclonal anti-CD68 (ab31630, dilution 1:200), rabbit polyclonal anti-CD206 (ab64693, dilution 1:800). Sections were then incubated with a ready-to-use anti-rabbit and anti-mouse secondary antibody labelled with peroxidase (Vector Laboratories, ref. MP-7401-50 and MP-7402-50), and a diaminobenzidine (DAB) substrate kit (Vector Laboratories, SK-4100) was used to reveal the immune reaction. Finally, samples were counterstained with Harry’s haematoxylin (Thermo Fisher Scientific) for 15 s followed by 3 min in tap water.

### 4.8. Statistical Analysis

For each variable analyzed in the study, we first determined the average and standard deviation for each global group and each specific group of samples. Global groups were: (1) all samples corresponding to each study time (24 and 48 h), (2) all samples corresponding to the same agarose type (all concentrations) at each study time, (3) all samples corresponding to the same agarose concentration (all agarose types) at each study time. Specific groups corresponded to a concrete concentration and a specific agarose type (for example, 3% concentration of agarose type D5) at each study time. Then, statistical comparisons among study groups were carried out with non-parametric statistics, since data followed a non-normal distribution. To detect significant differences among several study groups, Kruskal-Wallis test was used. Then, pair-wise comparisons between two groups, were performed by using the post-hoc test of Mann-Whitney. To determine the correlation between two variables Kendall’s Tau correlation test was applied. All data were analyzed with RealStatistics (Dr. Charles Zaiontz, Purdue University, West Lafayette, IN, USA), and *p* values below 0.05 were considered statistically significant for the double-tailed tests.

## Data Availability

The data presented in this study are available on request from the corresponding authors.

## Supplementary Materials

The following are available online at https://www.mdpi.com/1422-0067/22/4/1923/s1.
