# Supplementary material for: Evaluation of Marine Agarose Biomaterials for Tissue Engineering Applications
Source: Int J Mol Sci. 2021 Feb 15;22(4):1923. doi: 10.3390/ijms22041923 (PMC7919481; doi:10.3390/ijms22041923)
Supplement: Supplementary file 1 [file ijms-22-01923-s001.zip › Supplementary Table S1.docx]

| **Time** | **Agarose type** | **Agarose concentration** | **INDIRECT EFFECT OF AAH ON CELL VIABILITY AND FUNCTION** | | | **DIRECT EFFECT OF CAH ON CELL VIABILITY AND FUNCTION** | | | **BIOMECHANICAL PROPERTIES OF AGAROSE HYDROGELS** | | | |
| --- | --- | --- | --- | --- | --- | --- | --- | --- | --- | --- | --- | --- |
|  |  |  | **LIVE/DEAD (%)** | **WST-1 (%)** | **DNA (%)** | **LIVE/DEAD (%)** | **WST-1 (%)** | **DNA (%)** | **YOUNG MODULUS (Mpa)** | **STRESS AT FRACTURE (MPa)** | **STRAIN AT FRACTURE (mm/mm)** | **BREAK LOAD (N)** |
| **24 h** | **All** | **All** | 90.24 ± 4.5 | 76.6 ± 19.23 | 0 ± 0 | 90.68 ± 2.21 | 39.14 ± 9.38 | 1.13 ± 0.92 | 0.1086 ± 0.1405 | 0.0129 ± 0.0168 | 0.2747 ± 0.0932 | 25.4701 ± 33.1651 |
| **48 h** | **All** | **All** | 92.41 ± 5.82 | 87.28 ± 15.58 | 0.03 ± 0.13 | 94 ± 3.39 | 28.42 ± 5.36 | 0.15 ± 0.39 |  |  |  |  |
| **24 h** | **D1LE** | **AC** | 94.31 ± 1.97 | 73.2 ± 12.7 | 0 ± 0 | 84.2 ± 1.19 | 31.12 ± 14.82 | 1.94 ± 1.19 | 0.1152 ± 0.1611 | 0.014 ± 0.0195 | 0.3121 ± 0.1355 | 27.5663 ± 38.4511 |
| **48 h** | **D1LE** | **AC** | 93.28 ± 4.3 | 86.7 ± 11.29 | 0.16 ± 0.66 | 94.37 ± 1.72 | 9.18 ± 6.21 | 0.63 ± 1.62 |  |  |  |  |
| **24 h** | **D2LE** | **AC** | 91.44 ± 3.47 | 84.31 ± 19.98 | 0 ± 0 | 85.16 ± 2.05 | 32.5 ± 9.58 | 0.11 ± 0.34 | 0.1289 ± 0.1711 | 0.0144 ± 0.0194 | 0.2418 ± 0.0701 | 28.3526 ± 38.2771 |
| **48 h** | **D2LE** | **AC** | 88.32 ± 11.73 | 93.87 ± 12.17 | 0 ± 0 | 97.31 ± 1.98 | 41.67 ± 12.5 | 0 ± 0 |  |  |  |  |
| **24 h** | **LM** | **AC** | 94.16 ± 1.82 | 81.36 ± 24.3 | 0 ± 0 | 94.44 ± 1.01 | 35.2 ± 6.51 | 1.7 ± 1.1 | 0.0958 ± 0.0977 | 0.0075 ± 0.0068 | 0.2057 ± 0.0518 | 14.8737 ± 13.4067 |
| **48 h** | **LM** | **AC** | 97.73 ± 2.51 | 91.81 ± 17.43 | 0 ± 0 | 94.79 ± 4.28 | 50.64 ± 1.3 | 0 ± 0 |  |  |  |  |
| **24 h** | **MS8** | **AC** | 89.41 ± 6.1 | 44.15 ± 39.17 | 0 ± 0 | 98.12 ± 2.12 | 42.11 ± 6.6 | 0.55 ± 0.66 | 0.0814 ± 0.0997 | 0.0108 ± 0.0147 | 0.2694 ± 0.0439 | 21.2544 ± 29.0349 |
| **48 h** | **MS8** | **AC** | 91.72 ± 6.62 | 66.9 ± 29.62 | 0 ± 0 | 96.49 ± 2.95 | 1.37 ± 2.29 | 0 ± 0 |  |  |  |  |
| **24 h** | **D5** | **AC** | 81.89 ± 9.14 | 100 ± 0 | 0 ± 0 | 91.5 ± 4.69 | 54.78 ± 9.4 | 1.37 ± 1.28 | 0.1203 ± 0.1547 | 0.0164 ± 0.0184 | 0.3189 ± 0.0847 | 32.2189 ± 36.227 |
| **48 h** | **D5** | **AC** | 91.01 ± 3.94 | 97.11 ± 7.41 | 0 ± 0 | 87.06 ± 6.01 | 39.26 ± 4.52 | 0.14 ± 0.32 |  |  |  |  |
| **24 h** | **AT** | **0.3%** | 90.74 ± 3.42 | 89.01 ± 16.24 | 0 ± 0 | 99.03 ± 0.24 | 75.31 ± 12.73 | 1.69 ± 1.7 | 0.0041 ± 0.0016 | 0.0006 ± 0.0002 | 0.38 ± 0.0912 | 1.2717 ± 0.4223 |
| **48 h** | **AT** | **0.3%** | 93.94 ± 7.24 | 83.94 ± 19.12 | 0 ± 0 | 97.55 ± 0.43 | 64.51 ± 4.46 | 0.22 ± 0.43 |  |  |  |  |
| **24 h** | **AT** | **0.5%** | 90.62 ± 4.52 | 78.56 ± 15.16 | 0 ± 0 | 99 ± 0.92 | 51.99 ± 9.58 | 2.06 ± 1.14 | 0.0104 ± 0.0052 | 0.0014 ± 0.001 | 0.2838 ± 0.0652 | 2.9306 ± 2.0273 |
| **48 h** | **AT** | **0.5%** | 92.32 ± 6.39 | 84.96 ± 15.01 | 0 ± 0 | 97.37 ± 2.41 | 47.01 ± 12.43 | 0.01 ± 0.04 |  |  |  |  |
| **24 h** | **AT** | **1.0%** | 90.12 ± 4.99 | 80.37 ± 17.56 | 0 ± 0 | 97.11 ± 1.46 | 24.17 ± 12 | 0.78 ± 0.82 | 0.0621 ± 0.0239 | 0.008 ± 0.0049 | 0.2658 ± 0.079 | 15.8548 ± 9.6693 |
| **48 h** | **AT** | **1.0%** | 94.27 ± 3.21 | 90.21 ± 14.86 | 0.12 ± 0.53 | 98.61 ± 1.43 | 1.66 ± 3.52 | 0.38 ± 1.08 |  |  |  |  |
| **24 h** | **AT** | **3.0%** | 89.47 ± 5.06 | 58.48 ± 26.13 | 0 ± 0 | 67.6 ± 7.26 | 5.1 ± 3.21 | 0 ± 0 | 0.3185 ± 0.0857 | 0.037 ± 0.0129 | 0.2011 ± 0.0506 | 72.7405 ± 25.4084 |
| **48 h** | **AT** | **3.0%** | 89.11 ± 6.44 | 90 ± 10.43 | 0 ± 0 | 82.47 ± 9.28 | 0.51 ± 1.04 | 0 ± 0 |  |  |  |  |
| **24 h** | **D1LE** | **0.3%** | 95.32 ± 1.77 | 97.28 ± 5.78 | 0 ± 0 | 95.62 ± 0.77 | 73.22 ± 41.2 | 3.84 ± 3.14 | 0.0034 ± 0.0017 | 0.0004 ± 0.0001 | 0.501 ± 0.0392 | 0.7821 ± 0.1391 |
| **48 h** | **D1LE** | **0.3%** | 94.48 ± 4.38 | 92.79 ± 6.77 | 0 ± 0 | 99.64 ± 0.45 | 28.85 ± 12.62 | 1.08 ± 2.15 |  |  |  |  |
| **24 h** | **D1LE** | **0.5%** | 92.91 ± 1.91 | 99.94 ± 0.27 | 0 ± 0 | 100 ± 0 | 42.74 ± 13 | 3.82 ± 1.47 | 0.008 ± 0.0037 | 0.0008 ± 0.0002 | 0.2845 ± 0.0965 | 1.6371 ± 0.496 |
| **48 h** | **D1LE** | **0.5%** | 95.8 ± 1.35 | 94.12 ± 6.72 | 0 ± 0 | 100 ± 0 | 5.06 ± 3.74 | 0.07 ± 0.22 |  |  |  |  |
| **24 h** | **D1LE** | **1.0%** | 93.78 ± 2.62 | 68 ± 32.43 | 0 ± 0 | 100 ± 0 | 8.51 ± 5.1 | 0.09 ± 0.18 | 0.0682 ± 0.0215 | 0.008 ± 0.0027 | 0.2685 ± 0.0753 | 15.9008 ± 5.4339 |
| **48 h** | **D1LE** | **1.0%** | 93.52 ± 3.19 | 75.97 ± 17.89 | 0.62 ± 2.64 | 100 ± 0 | 2.82 ± 8.47 | 1.37 ± 4.11 |  |  |  |  |
| **24 h** | **D1LE** | **3.0%** | 95.22 ± 1.57 | 27.59 ± 12.33 | 0 ± 0 | 41.2 ± 3.35 | 0 ± 0 | 0 ± 0 | 0.3733 ± 0.0538 | 0.0458 ± 0.0001 | 0.1873 ± 0.0324 | 90.001 ± 0.0016 |
| **48 h** | **D1LE** | **3.0%** | 89.34 ± 8.29 | 83.95 ± 13.8 | 0 ± 0 | 77.87 ± 5.51 | 0 ± 0 | 0 ± 0 |  |  |  |  |
| **24 h** | **D2LE** | **0.3%** | 82.4 ± 23.96 | 88.69 ± 21.58 | 0 ± 0 | 100 ± 0 | 94.48 ± 6.43 | 0.38 ± 1.13 | 0.0037 ± 0.0009 | 0.0005 ± 0.0001 | 0.3015 ± 0.0184 | 1.1105 ± 0.1785 |
| **48 h** | **D2LE** | **0.3%** | 88.25 ± 21.52 | 88.1 ± 24.9 | 0 ± 0 | 100 ± 0 | 100 ± 0 | 0 ± 0 |  |  |  |  |
| **24 h** | **D2LE** | **0.5%** | 92.78 ± 5.61 | 91.77 ± 13.5 | 0 ± 0 | 96.46 ± 2.74 | 23.85 ± 17.62 | 0.02 ± 0.05 | 0.0067 ± 0.0045 | 0.0006 ± 0.0003 | 0.2682 ± 0.0593 | 1.3127 ± 0.7133 |
| **48 h** | **D2LE** | **0.5%** | 83.75 ± 19.73 | 92.06 ± 12.73 | 0 ± 0 | 95.99 ± 1.29 | 66.67 ± 50 | 0 ± 0 |  |  |  |  |
| **24 h** | **D2LE** | **1.0%** | 96.1 ± 1.76 | 98.31 ± 3.38 | 0 ± 0 | 89.87 ± 2.59 | 11.42 ± 13.71 | 0.07 ± 0.21 | 0.0524 ± 0.0116 | 0.0054 ± 0.0021 | 0.246 ± 0.0845 | 10.714 ± 4.2584 |
| **48 h** | **D2LE** | **1.0%** | 93.84 ± 2.07 | 99.03 ± 2.3 | 0 ± 0 | 98.33 ± 2.04 | 0 ± 0 | 0 ± 0 |  |  |  |  |
| **24 h** | **D2LE** | **3.0%** | 94.47 ± 3.04 | 58.49 ± 41.48 | 0 ± 0 | 54.31 ± 14.19 | 0.26 ± 0.59 | 0 ± 0 | 0.3903 ± 0.0252 | 0.0441 ± 0.0041 | 0.1814 ± 0.0386 | 86.6522 ± 8.2017 |
| **48 h** | **D2LE** | **3.0%** | 87.47 ± 3.63 | 96.33 ± 8.78 | 0 ± 0 | 94.91 ± 3.54 | 0 ± 0 | 0 ± 0 |  |  |  |  |
| **24 h** | **LM** | **0.3%** | 91.06 ± 2.64 | 93.42 ± 15.15 | 0 ± 0 | 100 ± 0 | 66.38 ± 9.76 | 1.95 ± 1.62 | - | - | - | - |
| **48 h** | **LM** | **0.3%** | 96.92 ± 3.54 | 93.8 ± 9.84 | 0 ± 0 | 99.93 ± 0.09 | 100 ± 0 | 0 ± 0 |  |  |  |  |
| **24 h** | **LM** | **0.5%** | 90.55 ± 3.21 | 65.31 ± 27.47 | 0 ± 0 | 99.02 ± 0.69 | 38 ± 4.32 | 3.23 ± 1.59 | 0.0064 ± 0.0018 | 0.0007 ± 0.0001 | 0.2192 ± 0.0262 | 1.5561 ± 0.3008 |
| **48 h** | **LM** | **0.5%** | 97.27 ± 1.88 | 83.64 ± 31.27 | 0 ± 0 | 99.08 ± 1.13 | 100 ± 0 | 0 ± 0 |  |  |  |  |
| **24 h** | **LM** | **1.0%** | 97.07 ± 1.06 | 91.28 ± 15.93 | 0 ± 0 | 100 ± 0 | 21.62 ± 8.22 | 1.64 ± 1.21 | 0.035 ± 0.0078 | 0.0043 ± 0.0014 | 0.2357 ± 0.033 | 8.554 ± 2.7843 |
| **48 h** | **LM** | **1.0%** | 99.04 ± 0.48 | 89.81 ± 28.63 | 0 ± 0 | 100 ± 0 | 0 ± 0 | 0 ± 0 |  |  |  |  |
| **24 h** | **LM** | **3.0%** | 97.98 ± 0.4 | 75.47 ± 41.84 | 0 ± 0 | 78.76 ± 2.8 | 14.81 ± 3.75 | 0 ± 0 | 0.2163 ± 0.0194 | 0.0153 ± 0.004 | 0.1668 ± 0.0594 | 30.0717 ± 8.0269 |
| **48 h** | **LM** | **3.0%** | 97.69 ± 4.13 | 100 ± 0 | 0 ± 0 | 80.16 ± 13.62 | 2.58 ± 5.18 | 0 ± 0 |  |  |  |  |
| **24 h** | **MS8** | **0.3%** | 94.7 ± 1.37 | 65.68 ± 38.71 | 0 ± 0 | 99.51 ± 0.3 | 42.47 ± 6.3 | 0.35 ± 0.57 | 0.0054 ± 0.002 | 0.0007 ± 0.0001 | 0.2975 ± 0.0185 | 1.5589 ± 0.269 |
| **48 h** | **MS8** | **0.3%** | 97.34 ± 4.43 | 50.58 ± 37.49 | 0 ± 0 | 99.35 ± 0.8 | 0 ± 0 | 0 ± 0 |  |  |  |  |
| **24 h** | **MS8** | **0.5%** | 81.46 ± 8.44 | 35.8 ± 46.89 | 0 ± 0 | 99.54 ± 0.57 | 61.23 ± 4.98 | 1.3 ± 1.47 | 0.0127 ± 0.0032 | 0.0019 ± 0.0005 | 0.2864 ± 0.0237 | 3.8423 ± 1.1529 |
| **48 h** | **MS8** | **0.5%** | 97.31 ± 2.22 | 59.6 ± 42.03 | 0 ± 0 | 95.54 ± 3.52 | 0 ± 0 | 0 ± 0 |  |  |  |  |
| **24 h** | **MS8** | **1.0%** | 92.42 ± 5.49 | 44.28 ± 36.07 | 0 ± 0 | 97.42 ± 1.59 | 54.3 ± 3.41 | 0.57 ± 0.61 | 0.069 ± 0.0133 | 0.0064 ± 0.0014 | 0.2213 ± 0.0594 | 12.6389 ± 2.9266 |
| **48 h** | **MS8** | **1.0%** | 94.52 ± 6.89 | 86.24 ± 25.51 | 0 ± 0 | 96.16 ± 2.38 | 5.46 ± 9.16 | 0 ± 0 |  |  |  |  |
| **24 h** | **MS8** | **3.0%** | 89.05 ± 9.12 | 30.86 ± 35.03 | 0 ± 0 | 96 ± 4.9 | 10.46 ± 11.7 | 0 ± 0 | 0.2386 ± 0.0559 | 0.0341 ± 0.0106 | 0.2722 ± 0.0189 | 66.9774 ± 20.9966 |
| **48 h** | **MS8** | **3.0%** | 77.74 ± 12.96 | 71.19 ± 25.86 | 0 ± 0 | 94.91 ± 3.54 | 0 ± 0 | 0 ± 0 |  |  |  |  |
| **24 h** | **D5** | **0.3%** | 90.26 ± 7.93 | 100 ± 0 | 0 ± 0 | 100 ± 0 | 100 ± 0 | 1.96 ± 2.06 | 0.0036 ± 0.0009 | 0.0007 ± 0.0001 | 0.3808 ± 0.0429 | 1.5548 ± 0.3322 |
| **48 h** | **D5** | **0.3%** | 92.75 ± 2.34 | 94.47 ± 16.63 | 0 ± 0 | 88.85 ± 0.51 | 93.7 ± 9.66 | 0 ± 0 |  |  |  |  |
| **24 h** | **D5** | **0.5%** | 95.43 ± 3.46 | 100 ± 0 | 0 ± 0 | 100 ± 0 | 94.13 ± 8.01 | 1.96 ± 1.16 | 0.0171 ± 0.0025 | 0.0029 ± 0.0007 | 0.339 ± 0.0434 | 5.8464 ± 1.4815 |
| **48 h** | **D5** | **0.5%** | 87.48 ± 6.77 | 95.39 ± 9.31 | 0 ± 0 | 96.3 ± 4.54 | 63.34 ± 8.41 | 0 ± 0 |  |  |  |  |
| **24 h** | **D5** | **1.0%** | 71.25 ± 14.01 | 100 ± 0 | 0 ± 0 | 98.25 ± 2.15 | 25 ± 29.59 | 1.57 ± 1.91 | 0.0868 ± 0.0251 | 0.016 ± 0.004 | 0.3582 ± 0.0669 | 31.4741 ± 8.0409 |
| **48 h** | **D5** | **1.0%** | 90.45 ± 3.46 | 100 ± 0 | 0 ± 0 | 98.55 ± 1.77 | 0 ± 0 | 0.56 ± 1.3 |  |  |  |  |
| **24 h** | **D5** | **3.0%** | 66.13 ± 15.74 | 100 ± 0 | 0 ± 0 | 67.76 ± 14.1 | 0 ± 0 | 0 ± 0 | 0.3739 ± 0.0439 | 0.0458 ± 0.0001 | 0.1977 ± 0.0156 | 90.0004 ± 0.0002 |
| **48 h** | **D5** | **3.0%** | 93.37 ± 3.21 | 98.58 ± 3.72 | 0 ± 0 | 64.54 ± 14.01 | 0 ± 0 | 0 ± 0 |  |  |  |  |

**SUPPLEMENTARY TABLE S1.** Average and standard deviation values obtained for each study group (global groups of samples with the same concentration or the same agarose type, and specific groups of samples of each agarose type and concentration). All: all agarose types and concentrations considered together; AC: all agarose concentrations; AT: all agarose types. For the cell viability and function, results are shown as percentages normalized to negative controls of dead cells (0% viability and function) and positive controls of live cells (100% viability and function).
