# Supplementary material for: Evaluation of Marine Agarose Biomaterials for Tissue Engineering Applications
Source: Int J Mol Sci. 2021 Feb 15;22(4):1923. doi: 10.3390/ijms22041923 (PMC7919481; doi:10.3390/ijms22041923)
Supplement: Supplementary file 1 [file ijms-22-01923-s001.zip › Supplementary Table S2.docx]

**SUPPLEMENTARY TABLE S2.** Statistical analysis of global study groups. To detect significant differences among several study groups analyzed with the same technique (for instance, break load for the five agarose types), we used the Kruskal-Wallis test, and specific comparison of two study groups (for instance, 1% vs. 3%) was carried out using Mann-Whitney tests. Global comparisons correspond to the analysis of all agarose concentrations or all agarose types. All agarose types within each concentration correspond to the comparison between two specific concentrations regardless the agarose type (all agarose types included), whereas all agarose concentrations within each agarose type correspond to the comparison between two specific agarose types regardless the agarose concentration (all agarose concentrations included). Among all agarose types within each concentration shows the results of the global comparison among the 5 agarose types regardless the concentration (all agarose concentrations included), whereas among all concentrations within each agarose type shows the results of the global comparison among the 4 agarose concentrations regardless the agarose type (all agarose types included). Statistically significant p values are labeled with asterisks (*).

|  | | **INDIRECT EFFECT OF AAH ON CELL VIABILITY AND FUNCTION** | | | | | | **DIRECT EFFECT OF CAH ON CELL VIABILITY AND FUNCTION** | | | | | | **BIOMECHANICAL PROPERTIES OF AGAROSE HYDROGELS** | | | |
| --- | --- | --- | --- | --- | --- | --- | --- | --- | --- | --- | --- | --- | --- | --- | --- | --- | --- |
|  |  | **LIVE/DEAD** | | **WST-1** | | **DNA** | | **LIVE/DEAD** | | **WST-1** | | **DNA** | |  |  |  |  |
|  |  | **24h** | **48h** | **24h** | **48h** | **24h** | **48h** | **24h** | **48h** | **24h** | **48h** | **24h** | **48h** | **YOUNG MODULUS** | **STRESS AT FRACTURE** | **STRAIN AT FRACTURE** | **BREAK LOAD (N)** |
| **Global comparison among all agarose concentrations** | | 0.4857 | 0.0253* | <0.0001* | 0.1625 | 1.0000 | 0.3916 | <0.0001* | <0.0001* | <0.0001* | <0.0001* | <0.0001* | 0.1561 | <0.0001* | <0.0001* | <0.0001* | <0.0001* |
| **All agarose types within each concentration** | **0.3% vs. 0.5%** | 0.6384 | 0.2131 | 0.0929 | 0.9079 | 1.0000 | 1.0000 | 0.6932 | 0.4315 | 0.0001* | 0.0567 | 0.1898 | 0.3091 | <0.0001* | 0.0004* | 0.0001* | 0.0004* |
|  | **0.3% vs. 1%** | 0.2958 | 0.4312 | 0.0184* | 0.0352* | 1.0000 | 0.3227 | 0.1764 | 0.4831 | <0.0001* | <0.0001* | 0.0517 | 0.6894 | <0.0001* | <0.0001* | <0.0001* | <0.0001* |
|  | **0.3% vs. 3%** | 0.3657 | 0.0064* | <0.0001* | 0.4274 | 1.0000 | 1.0000 | <0.0001* | <0.0001* | <0.0001* | <0.0001* | <0.0001* | 0.0821 | <0.0001* | <0.0001* | <0.0001* | <0.0001* |
|  | **0.5% vs. 1%** | 0.2131 | 0.769 | 0.6482 | 0.0654 | 1.0000 | 0.3227 | 0.2364 | 0.1709 | <0.0001* | <0.0001* | 0.0004* | 0.1732 | <0.0001* | <0.0001* | 0.2742 | <0.0001* |
|  | **0.5% vs. 3%** | 0.2904 | 0.0651 | 0.0002* | 0.5373 | 1.0000 | 1.0000 | <0.0001* | <0.0001* | <0.0001* | <0.0001* | <0.0001* | 0.3281 | <0.0001* | <0.0001* | <0.0001* | <0.0001* |
|  | **1% vs. 3%** | 0.8417 | 0.02* | 0.0003* | 0.1884 | 1.0000 | 0.3227 | <0.0001* | <0.0001* | <0.0001* | 0.3855 | <0.0001* | 0.0431* | <0.0001* | <0.0001* | 0.0015* | <0.0001* |
| **Among all agarose types within each concentration** | **0.3%** | 0.1754 | 0.0327* | 0.0032* | 0.0002* | 1.0000 | 1.0000 | 0.0002* | 0.0066* | <0.0001* | <0.0001* | 0.0005* | 0.0136* | 0.38 | 0.0052* | 0.0015* | 0.0053* |
|  | **0.5%** | 0.0073* | 0.0023* | <0.0001* | 0.0013* | 1.0000 | 1.0000 | 0.0069* | 0.0178* | <0.0001* | <0.0001* | <0.0001* | 0.406 | 0.0013* | 0.0003* | 0.0107* | 0.0003* |
|  | **1%** | 0.0005* | 0.0008* | <0.0001* | <0.0001* | 1.0000 | 0.406 | 0.0007* | 0.0159* | <0.0001* | 0.0675 | 0.0039* | 0.0675 | 0.001* | 0.0009* | 0.0375* | 0.0009* |
|  | **3%** | 0.0001* | 0.0011* | <0.0001* | <0.0001* | 1.0000 | 1.0000 | 0.0005* | 0.0041* | <0.0001* | 0.0851 | 1.0000 | 1.0000 | 0.0002* | 0.0001* | 0.0055* | 0.0048* |
| **Global comparison among all agarose types** | | 0.0011* | <0.0001* | <0.0001* | <0.0001* | 1.0000 | 0.406 | 0.2081 | 0.0344* | 0.0429* | <0.0001* | <0.0001* | 0.0077* | 0.9487 | 0.6549 | 0.0003* | 0.6757 |
| **All agarose concentrations within each agarose type** | **D1LE vs. D2LE** | 0.4175 | 0.1563 | 0.2088 | <0.0001* | 1.0000 | 0.324 | 0.3783 | 0.4264 | 0.9229 | 0.3202 | <0.0001* | 0.0221* | 0.76 | 0.8963 | 0.1209 | 0.9157 |
|  | **D1LE vs. LM** | 0.5388 | <0.0001* | 0.102 | <0.0001* | 1.0000 | 0.324 | 0.5759 | 0.1393 | 0.0684 | 0.0194* | 0.6147 | 0.0221* | 0.5776 | 0.4834 | 0.0289* | 0.4842 |
|  | **D1LE vs. MS8** | 0.0286* | 0.1138 | <0.0001* | 0.0189* | 1.0000 | 0.324 | 0.3661 | 0.0222* | 0.0149* | 0.0001* | 0.0473* | 0.0221* | 0.6626 | 0.4236 | 0.8899 | 0.3657 |
|  | **D1LE vs. D5** | 0.0029* | 0.0077* | <0.0001* | <0.0001* | 1.0000 | 0.324 | 0.3881 | 0.8241 | 0.114 | 0.0517 | 0.6668 | 0.4328 | 0.4758 | 0.1504 | 0.6937 | 0.1665 |
|  | **D2LE vs. LM** | 0.8936 | <0.0001* | 0.9663 | 0.4751 | 1.0000 | 1.0000 | 0.0335* | 0.3488 | 0.0491* | 0.3384 | <0.0001* | 1.0000 | 0.8661 | 0.6562 | 0.1009 | 0.6566 |
|  | **D2LE vs. MS8** | 0.0304* | 0.0324* | <0.0001* | <0.0001* | 1.0000 | 1.0000 | 0.0809 | 0.0233* | 0.047* | 0.0003* | 0.0043* | 1.0000 | 0.8824 | 0.7583 | 0.1191 | 0.7073 |
|  | **D2LE vs. D5** | 0.004* | 0.7617 | <0.0001* | 0.043* | 1.0000 | 1.0000 | 0.0573 | 0.4024 | 0.0789 | 0.6581 | <0.0001* | 0.0818 | 0.7674 | 0.3533 | 0.0025* | 0.381 |
|  | **LM vs. MS8** | 0.0447* | 0.0311* | <0.0001* | <0.0001* | 1.0000 | 1.0000 | 0.5799 | 0.0771 | 0.1227 | <0.0001* | 0.0009* | 1.0000 | 0.6687 | 0.9011 | 0.0001* | 0.9011 |
|  | **LM vs. D5** | 0.001* | <0.0001* | <0.0001* | 0.2248 | 1.0000 | 1.0000 | 0.6221 | 0.1099 | 0.2701 | 0.1269 | 0.3196 | 0.0818 | 0.7931 | 0.4636 | 0.0003* | 0.4644 |
|  | **MS8 vs. D5** | 0.093 | 0.0142* | <0.0001* | <0.0001* | 1.0000 | 1.0000 | 0.9162 | 0.0205* | 0.3055 | <0.0001* | 0.0287* | 0.0818 | 0.5706 | 0.3577 | 0.0075* | 0.4272 |
| **Among all concentrations within each agarose type** | **D1LE** | 0.0762 | 0.1432 | <0.0001* | 0.0009* | 1.0000 | 0.3916 | <0.0001* | 0.0001* | <0.0001* | <0.0001* | <0.0001* | 0.2532 | 0.0001* | 0.0001* | 0.0011* | 0.0001* |
|  | **D2LE** | 0.123 | 0.0712 | 0.0001* | 0.4104 | 1.0000 | 1.0000 | <0.0001* | 0.001* | <0.0001* | <0.0001* | 0.5582 | 1.0000 | 0.0005* | 0.0005* | 0.0442* | 0.0005* |
|  | **LM** | 0.0001* | 0.0908 | 0.0066* | 0.0472* | 1.0000 | 1.0000 | 0.0076* | 0.005* | <0.0001* | <0.0001* | <0.0001* | 1.0000 | 0.0013* | 0.0013* | 0.0984 | 0.0013* |
|  | **MS8** | 0.0208* | 0.005* | 0.0331* | 0.0066* | 1.0000 | 1.0000 | 0.0001* | 0.0002* | <0.0001* | 0.0001* | 0.0248* | 1.0000 | <0.0001* | <0.0001* | 0.039* | <0.0001* |
|  | **D5** | 0.0005* | 0.1298 | 1.0000 | 0.2399 | 1.0000 | 1.0000 | <0.0001* | 0.0011* | <0.0001* | <0.0001* | 0.0022* | 0.023* | <0.0001* | <0.0001* | 0.0026* | <0.0001* |
