# Supplementary material for: Evaluation of Marine Agarose Biomaterials for Tissue Engineering Applications
Source: Int J Mol Sci. 2021 Feb 15;22(4):1923. doi: 10.3390/ijms22041923 (PMC7919481; doi:10.3390/ijms22041923)
Supplement: Supplementary file 1 [file ijms-22-01923-s001.zip › Supplementary Table S3.docx]

**SUPPLEMENTARY TABLE S3.** Statistical analysis of specific study groups. Results correspond to the p values obtained for each analysis technique when two specific study groups (for instance, 0.3% D1LE vs. 0.3% D2LE) were compared using the Mann-Whitney test. Statistically significant p values are labeled with asterisks (*).

|  | | **INDIRECT EFFECT OF AAH ON CELL VIABILITY AND FUNCTION** | | | | | | **DIRECT EFFECT OF CAH ON CELL VIABILITY AND FUNCTION** | | | | | | **BIOMECHANICAL PROPERTIES OF AGAROSE HYDROGELS** | | | |
| --- | --- | --- | --- | --- | --- | --- | --- | --- | --- | --- | --- | --- | --- | --- | --- | --- | --- |
|  |  | **LIVE/DEAD** | **LIVE/DEAD** | **WST-1** | **WST-1** | **DNA** | **DNA** | **LIVE/DEAD** | **LIVE/DEAD** | **WST-1** | **WST-1** | **DNA** | **DNA** | **YOUNG MODULUS** | **STRESS AT FRACTURE** | **STRAIN AT FRACTURE** | **BREAK LOAD** |
|  |  | **24H** | **48H** | **24H** | **48H** | **24H** | **48H** | **24H** | **48H** | **24H** | **48H** | **24H** | **48H** |  |  |  |  |
| **0.3%** | **D1LE vs. D2LE** | 0.7768 | 0.3710 | 0.5221 | 0.1797 | 1.0000 | 1.0000 | 0.0072* | 0.0707 | 0.9227 | 0.0001* | 0.0772 | 0.0018* | 0.6985 | 0.0275* | 0.0281* | 0.0281* |
|  | **D1LE vs. LM** | 0.0072* | 0.0736 | 0.6637 | 0.1318 | 1.0000 | 1.0000 | 0.0116* | 0.6643 | 0.2420 | 0.0001* | 0.0772 | 0.2161 | 1.0000 | 1.0000 | 1.0000 | 1.0000 |
|  | **D1LE vs. MS8** | 0.3710 | 0.0298* | 0.0367* | 0.0289* | 1.0000 | 1.0000 | 0.0072* | 0.0189* | 0.1501 | 0.0001* | 0.0772 | 0.0063* | 0.2297 | 0.0049* | 0.005* | 0.005* |
|  | **D1LE vs. D5** | 0.3710 | 0.1252 | 0.0192* | 0.0017* | 1.0000 | 1.0000 | 0.0072* | 0.3855 | 0.0772 | 0.0003* | 0.0772 | 0.2158 | 0.5751 | 0.0049* | 0.005* | 0.005* |
|  | **D2LE vs. LM** | 0.9247 | 0.3710 | 0.2702 | 0.7773 | 1.0000 | 1.0000 | 0.0248* | 0.0707 | 0.0003* | 1.0000 | 1.0000 | 0.003* | 1.0000 | 1.0000 | 1.0000 | 1.0000 |
|  | **D2LE vs. MS8** | 0.9247 | 0.0736 | 0.1158 | 0.0052* | 1.0000 | 1.0000 | 1.0000 | 0.0107* | 0.0003* | <0.0001* | 1.0000 | 0.3955 | 0.1555 | 0.0518 | 0.8972 | 0.0528 |
|  | **D2LE vs. D5** | 0.9247 | 0.3066 | 0.0091* | 0.2887 | 1.0000 | 1.0000 | 1.0000 | 0.0707 | 0.0139* | 0.0772 | 1.0000 | 0.0062* | 1.0000 | 0.0932 | 0.0281* | 0.0932 |
|  | **LM vs. MS8** | 0.0106* | 0.2501 | 0.0142* | 0.0014* | 1.0000 | 1.0000 | 0.0248* | 0.0189* | 0.0004* | <0.0001* | 1.0000 | 0.009* | 1.0000 | 1.0000 | 1.0000 | 1.0000 |
|  | **LM vs. D5** | 1.0000 | 0.0552 | 0.0803 | 0.3282 | 1.0000 | 1.0000 | 0.0248* | 0.3855 | 0.0001* | 0.0772 | 1.0000 | 0.5960 | 1.0000 | 1.0000 | 1.0000 | 1.0000 |
|  | **MS8 vs. D5** | 0.5229 | 0.0409* | 0.0008* | 0.0003* | 1.0000 | 1.0000 | 1.0000 | 0.0189* | 0.0001* | 0.0001* | 1.0000 | 0.0228* | 0.0655 | 0.9360 | 0.005* | 0.9361 |
| **0.5%** | **D1LE vs. D2LE** | 0.7014 | 0.2013 | 0.0296* | 0.7910 | 1.0000 | 1.0000 | 0.0248* | 0.0072* | 0.0272* | 0.1897 | 0.3740 | 0.0002* | 0.3784 | 0.5211 | 0.9361 | 0.4711 |
|  | **D1LE vs. LM** | 0.2013 | 0.0966 | 0.0001* | 0.7371 | 1.0000 | 1.0000 | 0.0707 | 0.0248* | 0.3772 | 0.0001* | 0.3740 | 0.4797 | 0.5940 | 0.9151 | 0.1658 | 0.9151 |
|  | **D1LE vs. MS8** | 0.0298* | 0.1598 | 0.0001* | 0.001* | 1.0000 | 1.0000 | 1.0000 | 0.0707 | 0.0035* | 0.0005* | 0.3740 | 0.0131* | 0.0655 | 0.0082* | 0.3784 | 0.0082* |
|  | **D1LE vs. D5** | 0.0736 | 0.0151* | 0.3449 | 0.2618 | 1.0000 | 1.0000 | 0.0248* | 0.0707 | 0.0003* | 0.0004* | 0.3740 | 0.0267* | 0.005* | 0.005* | 0.0926 | 0.005* |
|  | **D2LE vs. LM** | 0.2501 | 0.0409* | 0.0034* | 0.8063 | 1.0000 | 1.0000 | 0.0886 | 0.6742 | 0.0151* | 0.0757 | 1.0000 | 0.0002* | 0.5940 | 0.7491 | 0.0699 | 0.7491 |
|  | **D2LE vs. MS8** | 0.0214* | 0.0214* | 0.0024* | 0.0055* | 1.0000 | 1.0000 | 0.0248* | 0.5270 | 0.001* | 0.0042* | 1.0000 | 0.012* | 0.0306* | 0.005* | 0.3784 | 0.005* |
|  | **D2LE vs. D5** | 0.3710 | 0.7014 | 0.0091* | 0.4585 | 1.0000 | 1.0000 | 0.1138 | 0.0114* | 0.0003* | 0.2412 | 1.0000 | 0.0009* | 0.005* | 0.005* | 0.0306* | 0.005* |
|  | **LM vs. MS8** | 0.0966 | 0.6092 | 0.0211* | 0.0109* | 1.0000 | 1.0000 | 0.0707 | 0.6704 | 0.0004* | <0.0001* | 1.0000 | 0.0269* | 0.0251* | 0.0142* | 0.0142* | 0.0142* |
|  | **LM vs. D5** | 0.0298* | 0.0032* | <0.0001* | 0.3890 | 1.0000 | 1.0000 | 0.2873 | 0.0886 | 0.0003* | 0.0001* | 1.0000 | 0.0769 | 0.0142* | 0.0142* | 0.0142* | 0.0142* |
|  | **MS8 vs. D5** | 0.0049* | 0.0049* | <0.0001* | 0.0004* | 1.0000 | 1.0000 | 0.0248* | 0.3855 | 0.0003* | 0.0001* | 1.0000 | 0.2664 | 0.0453* | 0.0306* | 0.0306* | 0.0306* |
| **1%** | **D1LE vs. D2LE** | 0.0736 | 1.0000 | 0.0017* | <0.0001* | 1.0000 | 0.3449 | 0.0072* | 0.0707 | 1.0000 | 0.3740 | 0.3740 | 0.9516 | 0.2353 | 0.0828 | 0.6480 | 0.0828 |
|  | **D1LE vs. LM** | 0.0106* | 0.0032* | 0.0156* | 0.0002* | 1.0000 | 0.3449 | 0.0248* | 0.0248* | 0.0019* | 0.3740 | 0.3740 | 0.0026* | 0.0081* | 0.0137* | 0.6480 | 0.0137* |
|  | **D1LE vs. MS8** | 0.6092 | 0.3066 | 0.016* | 0.0131* | 1.0000 | 0.3449 | 0.0707 | 0.0707 | 0.0004* | 0.3960 | 0.3740 | 0.0702 | 0.9272 | 0.4113 | 0.4113 | 0.4113 |
|  | **D1LE vs. D5** | 0.0032* | 0.0966 | <0.0001* | <0.0001* | 1.0000 | 0.3449 | 1.0000 | 1.0000 | 0.0849 | 0.3740 | 0.3960 | 0.0704 | 0.3153 | 0.0224* | 0.0828 | 0.0224* |
|  | **D2LE vs. LM** | 0.3066 | 0.0021* | 0.4265 | 0.8454 | 1.0000 | 1.0000 | 0.0116* | 0.2016 | 0.017* | 1.0000 | 1.0000 | 0.0019* | 0.0306* | 0.5751 | 0.8101 | 0.5751 |
|  | **D2LE vs. MS8** | 0.2501 | 0.1598 | <0.0001* | 0.2771 | 1.0000 | 1.0000 | 0.0114* | 0.6643 | 0.0004* | 0.0772 | 1.0000 | 0.0875 | 0.0655 | 0.4711 | 0.6889 | 0.4711 |
|  | **D2LE vs. D5** | 0.0021* | 0.0552 | 0.0192* | 0.0803 | 1.0000 | 1.0000 | 0.0072* | 0.0707 | 0.1449 | 1.0000 | 0.0772 | 0.0706 | 0.0082* | 0.005* | 0.0655 | 0.005* |
|  | **LM vs. MS8** | 0.2013 | 0.0409* | 0.0004* | 0.5143 | 1.0000 | 1.0000 | 0.3946 | 0.2016 | 0.0004* | 0.0772 | 1.0000 | 0.0444* | 0.005* | 0.0453* | 0.3784 | 0.0453* |
|  | **LM vs. D5** | 0.0021* | 0.0021* | 0.0091* | 0.0803 | 1.0000 | 1.0000 | 0.0248* | 0.0248* | 0.5958 | 1.0000 | 0.0772 | 0.5923 | 0.005* | 0.005* | 0.005* | 0.005* |
|  | **MS8 vs. D5** | 0.0072* | 0.0552 | <0.0001* | 0.0192* | 1.0000 | 1.0000 | 0.0707 | 0.0707 | 0.0061* | 0.0772 | 0.0772 | 0.4053 | 0.1734 | 0.005* | 0.013* | 0.005* |
| **3%** | **D1LE vs. D2LE** | 0.5229 | 0.3066 | 0.0846 | 0.0005* | 1.0000 | 1.0000 | 0.1400 | 0.0116* | 0.1693 | 1.0000 | 1.0000 | 1.0000 | 0.6889 | 0.4046 | 0.4711 | 0.6303 |
|  | **D1LE vs. LM** | 0.0021* | 0.0151* | 0.004* | <0.0001* | 1.0000 | 1.0000 | 0.0111* | 0.0116* | 0.0001* | 0.1693 | 1.0000 | 1.0000 | 0.005* | 0.0027* | 0.6889 | 0.005* |
|  | **D1LE vs. MS8** | 0.2013 | 0.0966 | 0.4764 | 0.1202 | 1.0000 | 1.0000 | 0.0114* | 0.1412 | 0.0018* | 1.0000 | 1.0000 | 1.0000 | 0.005* | 0.0284* | 0.005* | 0.2979 |
|  | **D1LE vs. D5** | 0.0021* | 0.3710 | <0.0001* | <0.0001* | 1.0000 | 1.0000 | 0.0111* | 0.5283 | 1.0000 | 1.0000 | 1.0000 | 1.0000 | 0.9361 | 1.0000 | 0.3784 | 0.7483 |
|  | **D2LE vs. LM** | 0.0298* | 0.0049* | 0.0243* | 0.0192* | 1.0000 | 1.0000 | 0.0114* | 0.9133 | 0.0002* | 0.1693 | 1.0000 | 1.0000 | 0.005* | 0.0036* | 0.8101 | 0.005* |
|  | **D2LE vs. MS8** | 0.2501 | 0.0966 | 0.2225 | 0.0002* | 1.0000 | 1.0000 | 0.2933 | 0.0116* | 0.0062* | 1.0000 | 1.0000 | 1.0000 | 0.005* | 0.0898 | 0.013* | 0.2979 |
|  | **D2LE vs. D5** | 0.0021* | 0.0151* | <0.0001* | 0.4339 | 1.0000 | 1.0000 | 0.0114* | 0.1138 | 0.1693 | 1.0000 | 1.0000 | 1.0000 | 0.5751 | 0.4046 | 0.0926 | 0.9361 |
|  | **LM vs. MS8** | 0.0214* | 0.0049* | 0.0368* | <0.0001* | 1.0000 | 1.0000 | 0.0203* | 0.0116* | 0.2505 | 0.1693 | 1.0000 | 1.0000 | 0.3784 | 0.0081* | 0.005* | 0.0082* |
|  | **LM vs. D5** | 0.0021* | 0.0298* | 0.0042* | 0.0803 | 1.0000 | 1.0000 | 0.0111* | 0.1138 | 0.0001* | 0.1693 | 1.0000 | 1.0000 | 0.005* | 0.0027* | 0.5751 | 0.005* |
|  | **MS8 vs. D5** | 0.0106* | 0.0298* | <0.0001* | <0.0001* | 1.0000 | 1.0000 | 0.1400 | 0.2933 | 0.0018* | 1.0000 | 1.0000 | 1.0000 | 0.005* | 0.0284* | 0.005* | 0.3784 |
| **D1LE** | **0.3% vs. 0.5%** | 0.0298* | 1.0000 | 0.0640 | 0.2168 | 1.0000 | 1.0000 | 0.0072* | 0.0707 | 0.1501 | 0.0004* | 1.0000 | 0.2751 | 0.0202* | 0.0049* | 0.0082* | 0.005* |
|  | **0.3% vs. 1%** | 0.3066 | 0.4432 | 0.0027* | 0.0099* | 1.0000 | 0.3449 | 0.0072* | 0.0707 | 0.0053* | 0.0008* | 0.0026* | 0.3960 | 0.0081* | 0.0079* | 0.0081* | 0.0081* |
|  | **0.3% vs. 3%** | 0.8983 | 0.1598 | <0.0001* | 0.0375* | 1.0000 | 1.0000 | 0.0114* | 0.0114* | 0.0004* | 0.0001* | 0.0005* | 0.0772 | 0.005* | 0.0027* | 0.005* | 0.005* |
|  | **0.5% vs. 1%** | 0.3710 | 0.1252 | 0.0001* | 0.0015* | 1.0000 | 0.3449 | 1.0000 | 1.0000 | 0.0004* | 0.0108* | 0.0002* | 1.0000 | 0.0081* | 0.0081* | 0.9272 | 0.0081* |
|  | **0.5% vs. 3%** | 0.0298* | 0.0409* | <0.0001* | 0.003* | 1.0000 | 1.0000 | 0.007* | 0.0072* | 0.0001* | 0.0005* | 0.0001* | 0.3740 | 0.005* | 0.0027* | 0.0202* | 0.005* |
|  | **1% vs. 3%** | 0.3066 | 0.3710 | 0.0005* | 0.3309 | 1.0000 | 0.3449 | 0.007* | 0.0072* | 0.0001* | 0.3740 | 0.1685 | 0.3740 | 0.0081* | 0.0038* | 0.0828 | 0.0081* |
| **D2LE** | **0.3% vs. 0.5%** | 0.1580 | 0.2501 | 0.9248 | 0.3965 | 1.0000 | 1.0000 | 0.0248* | 0.0072* | 0.0003* | 0.0757 | 1.0000 | 1.0000 | 0.6985 | 0.8972 | 0.2452 | 0.8972 |
|  | **0.3% vs. 1%** | 0.0542 | 0.2013 | 0.4043 | 0.5221 | 1.0000 | 1.0000 | 0.0072* | 0.0707 | 0.0003* | <0.0001* | 0.6834 | 1.0000 | 0.0281* | 0.0281* | 0.3661 | 0.0281* |
|  | **0.3% vs. 3%** | 0.0952 | 0.0552 | 0.0018* | 0.5477 | 1.0000 | 1.0000 | 0.0072* | 0.0248* | 0.0002* | <0.0001* | 0.3740 | 1.0000 | 0.0281* | 0.0162* | 0.0281* | 0.0281* |
|  | **0.5% vs. 1%** | 0.4432 | 0.2501 | 0.4153 | 0.1397 | 1.0000 | 1.0000 | 0.0207* | 0.0916 | 0.0930 | 0.0042* | 0.5866 | 1.0000 | 0.005* | 0.005* | 0.5751 | 0.005* |
|  | **0.5% vs. 3%** | 0.7982 | 0.7014 | 0.0022* | 0.1397 | 1.0000 | 1.0000 | 0.0116* | 0.4005 | 0.0014* | 0.0042* | 0.3740 | 1.0000 | 0.005* | 0.0036* | 0.0202* | 0.005* |
|  | **1% vs. 3%** | 0.2013 | 0.0151* | <0.0001* | 0.9398 | 1.0000 | 1.0000 | 0.0116* | 0.0886 | 0.0019* | 1.0000 | 0.1693 | 1.0000 | 0.005* | 0.0036* | 0.2297 | 0.005* |
| **LM** | **0.3% vs. 0.5%** | 0.7014 | 0.7982 | 0.0013* | 0.0803 | 1.0000 | 1.0000 | 0.3946 | 0.0886 | 0.0004* | 1.0000 | 0.0699 | 1.0000 | - | - | - | - |
|  | **0.3% vs. 1%** | 0.0021* | 0.0736 | 0.2702 | 0.0091* | 1.0000 | 1.0000 | 0.1138 | 0.0886 | 0.0004* | <0.0001* | 0.9647 | 1.0000 | - | - | - | - |
|  | **0.3% vs. 3%** | 0.0021* | 0.1598 | 0.1382 | 0.0091* | 1.0000 | 1.0000 | 0.5232 | 0.0886 | 0.0004* | <0.0001* | 0.0001* | 1.0000 | - | - | - | - |
|  | **0.5% vs. 1%** | 0.0021* | 0.0409* | 0.0064* | 0.2702 | 1.0000 | 1.0000 | 0.0886 | 0.9160 | 0.0006* | <0.0001* | 0.0378* | 1.0000 | 0.0142* | 0.0142* | 0.5940 | 0.0142* |
|  | **0.5% vs. 3%** | 0.0021* | 0.1252 | 0.1978 | 0.3282 | 1.0000 | 1.0000 | 0.3855 | 0.3427 | 0.0004* | <0.0001* | 0.0001* | 1.0000 | 0.0142* | 0.0142* | 0.2409 | 0.0142* |
|  | **1% vs. 3%** | 0.1598 | 0.8983 | 0.4961 | 0.7485 | 1.0000 | 1.0000 | 0.8314 | 0.6723 | 0.0337* | 0.1693 | 0.0005* | 1.0000 | 0.005* | 0.005* | 0.0453* | 0.005* |
| **MS8** | **0.3% vs. 0.5%** | 0.0032* | 0.3710 | 0.0172* | 0.0088* | 1.0000 | 1.0000 | 1.0000 | 0.0207* | 0.0004* | 1.0000 | 0.1568 | 1.0000 | 0.005* | 0.0049* | 0.3784 | 0.005* |
|  | **0.3% vs. 1%** | 0.5229 | 0.2013 | 0.1141 | 1.0000 | 1.0000 | 1.0000 | 0.0707 | 0.0116* | 0.0012* | 0.0772 | 0.4968 | 1.0000 | 0.005* | 0.0049* | 0.0453* | 0.005* |
|  | **0.3% vs. 3%** | 0.2501 | 0.0049* | 0.0085* | 0.2391 | 1.0000 | 1.0000 | 0.0072* | 0.1424 | 0.0004* | 1.0000 | 0.0768 | 1.0000 | 0.005* | 0.0049* | 0.0655 | 0.005* |
|  | **0.5% vs. 1%** | 0.0298* | 0.5229 | 0.2712 | 0.0032* | 1.0000 | 1.0000 | 0.0707 | 0.6643 | 0.0165* | 0.0772 | 0.3861 | 1.0000 | 0.005* | 0.005* | 0.0655 | 0.005* |
|  | **0.5% vs. 3%** | 0.1252 | 0.0072* | 0.5340 | 0.0024* | 1.0000 | 1.0000 | 0.0072* | 0.0114* | 0.0004* | 1.0000 | 0.0053* | 1.0000 | 0.005* | 0.0049* | 0.3784 | 0.005* |
|  | **1% vs. 3%** | 0.6092 | 0.0151* | 0.3102 | 0.8736 | 1.0000 | 1.0000 | 0.0114* | 0.0114* | 0.0004* | 0.0772 | 0.0138* | 1.0000 | 0.005* | 0.0049* | 0.0655 | 0.005* |
| **D5** | **0.3% vs. 0.5%** | 0.3710 | 0.0552 | 1.0000 | 0.0803 | 1.0000 | 1.0000 | 0.0248* | 0.3855 | 0.0139* | 0.0003* | 0.8249 | 1.0000 | 0.005* | 0.005* | 0.1282 | 0.005* |
|  | **0.3% vs. 1%** | 0.0106* | 0.3066 | 1.0000 | 0.5214 | 1.0000 | 1.0000 | 1.0000 | 0.0707 | 0.0001* | 0.0001* | 0.3722 | 0.0772 | 0.005* | 0.005* | 0.4711 | 0.005* |
|  | **0.3% vs. 3%** | 0.0106* | 0.5229 | 1.0000 | 0.9611 | 1.0000 | 1.0000 | 0.007* | 0.0886 | <0.0001* | 0.0001* | 0.0005* | 1.0000 | 0.005* | 0.0027* | 0.005* | 0.005* |
|  | **0.5% vs. 1%** | 0.0032* | 0.3710 | 1.0000 | 0.0395* | 1.0000 | 1.0000 | 0.0248* | 0.0707 | 0.0018* | 0.0001* | 0.2835 | 0.0772 | 0.005* | 0.005* | 0.8101 | 0.005* |
|  | **0.5% vs. 3%** | 0.0021* | 0.0966 | 1.0000 | 0.0803 | 1.0000 | 1.0000 | 0.0114* | 0.0886 | 0.0001* | 0.0001* | 0.0005* | 1.0000 | 0.005* | 0.0027* | 0.005* | 0.005* |
|  | **1% vs. 3%** | 0.3710 | 0.2013 | 1.0000 | 0.7659 | 1.0000 | 1.0000 | 0.007* | 0.0248* | 0.0005* | 1.0000 | 0.0139* | 0.0772 | 0.005* | 0.0027* | 0.005* | 0.005* |
