# Supplementary material for: Evaluation of Marine Agarose Biomaterials for Tissue Engineering Applications
Source: Int J Mol Sci. 2021 Feb 15;22(4):1923. doi: 10.3390/ijms22041923 (PMC7919481; doi:10.3390/ijms22041923)
Supplement: Supplementary file 1 [file ijms-22-01923-s001.zip › Supplementary Table S4.docx]

**SUPPLEMENTARY TABLE S4.** Materials and reagents used in the present work.

| **MATERIALS USED** | **COMPANY** | **REFERENCE** |
| --- | --- | --- |
| **Phosphate-Buffered Saline (PBS)** | **Sigma-Aldrich, St Louis, MO, USA** | **D8662** |
| **Type-I Collagenase** | **Sigma-Aldrich, St Louis, MO, USA** | **1148089** |
| **Dulbecco’s Modified Eagle Medium (Dmem)** | **Sigma-Aldrich, St Louis, MO, USA** | **D6429** |
| **Antibiotic Cocktail Solution** | **Sigma-Aldrich, St Louis, MO, USA** | **A5955** |
| **Fetal Bovine Serum** | **Sigma-Aldrich, St Louis, MO, USA** | **F7524** |
| **Agarose** | **Hispanagar, Burgos, Spain** | **D1LE, D2LE, LM, MS8 and D5** |
| **Instron Biomechanical Analyzer** | **Norwood, MA, USA** | **5943** |
| **6.5 mm-Diameter Porous Inserts** | **Corning, New York, NY, USA** | **CLS3470** |
| **LIVE/DEAD (LD) Cell Viability/Cytotoxicity Analysis Kit** | **Life Technologies, Carlsbad, CA, USA** | **L3224** |
| **Light Microscopy** | **Nikon, Tokyo, Japan** | **Eclipse 90i** |
| **Cell Proliferation Reagent Wst-1** | **Sigma Aldrich, St Louis, MO, USA** | **11644807001** |
| **Microplate Reader** | **Biochrom, Cambridge, UK** | **ASYS UVM340** |
| **UV-Vis Nanodrop Equipment** | **Thermo Fisher Scientific, Waltham, MA, USA** | **ND-2000** |
| **Triton X-100** | **Sigma Aldrich, St Louis, MO, USA** | **T8787** |
| **24-Well Culture Plates** | **Corning, New York, NY, USA** | **CLS3527** |
| **Ketamine** | **Boehringer Ingelheim, Ingelheim am Rhein, Germany** | **AF34498** |
| **Acepromazine** | **Boehringer Ingelheim, Ingelheim am Rhein, Germany** | **578293** |
| **Sysmex Automatic Hematological Analyzer** | **Roche, Basel, Switzerland** | **KX-21N** |
| **Clinical Chemistry Analyzer** | **Roche, Basel, Switzerland** | **Cobas C311** |
| **Buffered Formalin** | **Panreac Química S.L.U., Barcelona, Spain** | **252931.1214** |
| **Paraffin** | **Panreac Química S.L.U., Barcelona, Spain** | **143209** |
| **Sirius Red F3B** | **Sigma Aldrich, St Louis, MO, USA** | **365548** |
| **Ph 8 EDTA** | **Sigma Aldrich, St Louis, MO, USA** | **E5134** |
| **Ph 6 Citrate** | **Sigma Aldrich, St Louis, MO, USA** | **C8532** |
| **H_2_o_2_** | **Panreac Química S.L.U., Barcelona, Spain** | **1077.1211** |
| **Casein** | **Vector laboratories, Burlingame, CA, USA** | **SP5020** |
| **Normal Horse Serum** | **Vector laboratories, Burlingame, CA, USA** | **30022** |
| **Rabbit Monoclonal Anti-CD4** | **Abcam, Cambridge, UK** | **ab237722** |
| **Rabbit Monoclonal Anti-CD8** | **Abcam, Cambridge, UK** | **ab237709** |
| **Mouse Monoclonal Anti-CD68** | **Abcam, Cambridge, UK** | **ab31630** |
| **Rabbit Polyclonal Anti-CD206** | **Abcam, Cambridge, UK** | **ab64693** |
| **Ready-To-Use Anti-Rabbit Secondary Antibody Labelled With Peroxidase** | **Vector laboratories, Burlingame, CA, USA** | **MP-7401-50** |
| **Ready-To-Use Anti-Mouse Secondary Antibody Labelled With Peroxidase** | **Vector laboratories, Burlingame, CA, USA** | **MP-7402-50** |
| **Diaminobenzidine (DAB) Substrate Kit** | **Vector laboratories, Burlingame, CA, USA** | **SK-4100** |
| **Harry’s Haematoxylin** | **Thermo Fisher Scientific, Waltham, MA, USA** | **6765002** |
